# Relationship Between Sulcal Characteristics and Brain Aging

**DOI:** 10.3389/fnagi.2018.00339

**Published:** 2018-11-12

**Authors:** Kaide Jin, Tianqi Zhang, Marnie Shaw, Perminder Sachdev, Nicolas Cherbuin

**Affiliations:** ^1^Centre for Research on Ageing, Health and Wellbeing, The Australian National University, Canberra, ACT, Australia; ^2^Centre for Healthy Brain Ageing, School of Psychiatry, University of New South Wales, Sydney, NSW, Australia

**Keywords:** aging, brain atrophy, cortex, sulci, MRI

## Abstract

This study aimed to determine whether sulcal morphology differs between middle age (MA) and older healthy individuals. Furthermore, we sought to determine whether age-related differences in sulcal characteristics were more strongly associated with differences in local or global cortical volumes. Participants (age 44–50, *N* = 403; age 64–70, *N* = 390) from the Personality and Total Health Through Life (PATH) study were included. Sulci were 17.3% wider, on average, in old age (OA) compared to MA participants, with the largest difference in the left superior frontal sulcus. Differences in sulcal width were generally higher in males than females. Differences in the width of the superior frontal and central sulci were significantly associated with differences in the volume of adjacent local gyri, while age-related differences in the width of lateral and superior temporal sulci were associated with differences in whole brain cortical volume. These findings suggest that sulcal characteristics provide unique information about changes in local and global brain structure in aging.

## Introduction

Biological aging is associated with brain atrophy at both the micro and macroscopic scales (Esiri, 2007). At the microscopic level, neuronal death, shrinkage of dendritic trees and neuropil, as well as decrease in spine numbers are involved (Murphy et al., 1996; Kolb and Whishaw, 1998; Anderton, 2002). At the macroscopic level, neuroimaging studies show that volumetric decreases occur across the whole brain with some regions more affected than others (DeCarli et al., 2005; Fotenos et al., 2005).

Volumetric studies have been very effective in using magnetic resonance imaging (MRI) data to characterize localized patterns of cerebral atrophy across the lifespan. However, they also have some important limitations. Specifically, as MRI intensity contrast between gray matter (GM) and white matter (WM) decreases with age, estimates of cerebral atrophy tend to underestimate the actual rate of shrinkage (Kochunov et al., 2005; Lemaitre et al., 2012). Moreover, volumetric measures are not very sensitive to complicated brain surface folding and thus may introduce regional bias and decrease statistical power (Lemaitre et al., 2012).

Alternative measurements—which do not suffer from these limitations—could be useful to complement existing volumetric measures. Recently, the measure of sulcal morphology has become a more accessible approach to investigate neuroanatomical variability (Mangin et al., 2004b). Since sulcal measures are not dependent on the accurate identification of GM and WM borders, and are more sensitive to complex folding of the cerebral surface, they may be more sensitive to detecting age-related change in cerebral structure (Lamont et al., 2014).

With aging, sulci become wider and shallower (Rettmann et al., 2006; Liu et al., 2013a). Sulcal changes are the product of shrinkage in the gyri adjacent to them as well as more distal changes which may affect the brain’s global shape with many regional consequences. These global forces are driven by the combined changes in cortical GM and WM, as well as in other subcortical structures (Im et al., 2008; Kochunov et al., 2008; Liu et al., 2013b). However, a number of questions remain unanswered. First, little is known about the magnitude and localization of sulcal changes in middle-age (MA). Second, it is unclear whether sulcal changes are driven by atrophy of adjacent local structures or by change in global tissue volumes. Third, it is not clear whether sulcal width or depth is more strongly associated with brain structural differences.

The aim of this study is to address these questions by: (1) investigating how sulcal morphology differs between middle adulthood and old-age (OA); (2) determining whether the volume of local brain structures or the whole brain are more strongly associated with sulcal characteristics; and (3) investigating whether sulcal width or depth is more strongly associated with local brain volumetric differences. It was predicted that: (a) sulci in MA would be narrower and deeper than in OA; (b) local structural characteristics would be more strongly associated with sulcal morphology in most sulci than global volumetric measures; and (c) sulcal width would be more strongly associated with local brain volumetric differences than sulcal depth.

## Materials and Methods

### Participants

Participants were selected from the Personality and Total Health Through Life (PATH) project (Anstey et al., 2012). PATH participants were residents of the Australian Capital Territory and neighboring Queanbeyan, Australia, and were randomly recruited through the electoral roll (Anstey et al., 2012). Enrolment to vote is compulsory for Australian citizens, making this cohort representative of the population. All participants provided written informed consent and this study was carried out in accordance with the recommendations of the 2007 National Statement on Ethical Conduct in Human Research, the National Health Research Council (NHMRC). The protocol was approved by the Australian National University Ethics Committee and all participants provided written informed consent.

**Figure 1:**
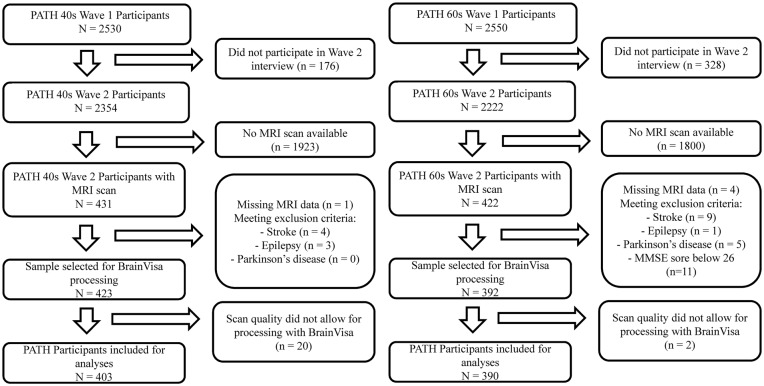
Sample inclusion (left: 40+ cohort; right: 60+ cohort).

The present study focuses on the MA (44–48 years) and the OA participants (64–68 years) at the second assessment of the PATH study, as MRI scans were first available at this wave for the MA sample. The selection process is summarized in Figure 1. Briefly, of participants initially included in PATH (40 s: *n* = 2530; 60 s: *n* = 2551) a sub-sample had an MRI scan at second assessment (40 s: *n* = 431; 60 s: *n* = 422). Of those, participants were excluded if they had a stroke, epilepsy, or Parkinson’s disease (40 s: *n* = 7; 60 s: *n* = 26; see Figure 1). The final sample includes 403 MA participants and 390 OA participants.

### MRI Acquisition

All participants were imaged with a 1.5-T Phillips Gyroscan ACS-NT scanner (Phillips Medical Systems, Best, Netherlands). T1-weighted 3-D structural images were acquired in coronal orientation using a fast-field echo sequence with the following parameters: repetition time/echo time = 28.005/2.64 ms; flip angle = 30°; matrix size = 256 × 256; field of view = 260 × 260 mm; slice thickness = 2.0 mm and mid-slice to mid-slice distance = 1.0 mm, providing over-contiguous coronal slices and an in-plane spatial resolution of 1.016 × 1.016 × 2 mm.

### MRI Analyses

All participants’ MRI scans were first processed using FreeSurfer^1^ (Fischl, 2012). FreeSurfer software enables the automatic parcellation of the volumes of the cortical surface using T1-weighted images (Dale et al., 1999). Briefly, this involved: (1) non-uniformity correction; (2) image registration; (3) skull stripping; (4) GM/WM segmentation; (5) surface corrections; and (6) parcellation of gyri (Dale et al., 1999; Desikan et al., 2006).

The three-dimensional cortical surface produced in Freesurfer was imported into BrainVISA for sulcal measurements^2^ (Shokouhi et al., 2011). A model of cortical sulci was automatically produced for each participant with the standard pipeline. Briefly, this included: (1) cortical surface extraction; (2) segmentation of gyral and sulcal regions; (3) computing a sulcal depth map; and (4) construction of a skeleton representing the shape or “hull” of identified sulci. Based on these steps, BrainVISA generates an integrated map combining all measurable sulci with labels extracted from a brain atlas (i.e., labels identified in FreeSurfer; Kochunov et al., 2012) and computes a measure of sulcal depth and width. Average sulcal width is defined as the average span of the intra-sulcal space along the normal projections to the sulcal mesh, i.e., the mean value of distances between two adjacent gyri along a sulcus. Average sulcal depth refers to the mean distance from the cortical surface of adjoining gyri to the deepest point in the sulcus.

### Regions of Interest (ROIs)

#### Sulcal ROIs

**Figure 2:**
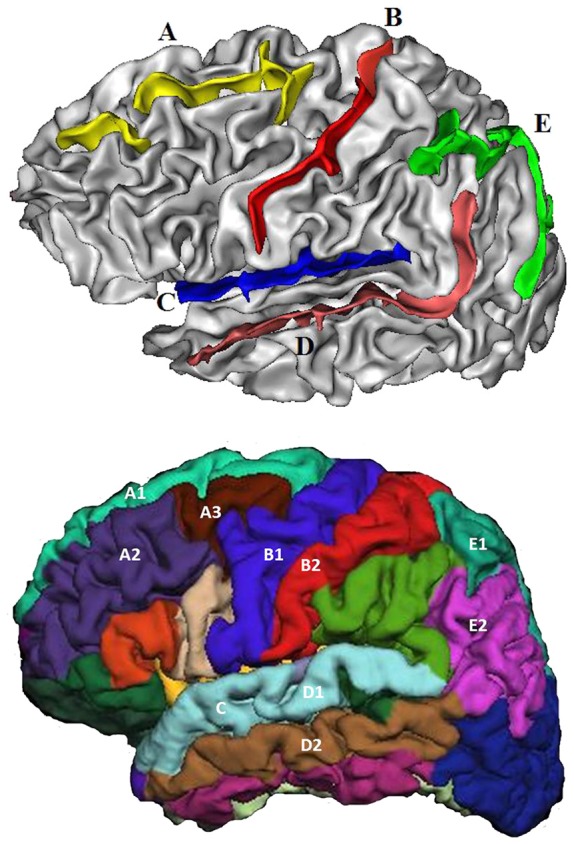
The five sulci and adjoining gyri selected for investigation. Top: **(A)** Superior frontal sulcus, **(B)** Central sulcus, **(C)** Lateral sulcus, **(D)** Superior temporal sulcus, and **(E)** Intra-parietal sulcus. Bottom: Superior frontal sulcus: superior frontal **(A1)**, middle frontal **(A2)** + **(A3)**. Central sulcus: pre-central **(B1)**, post-central **(B2)**. Lateral sulcus: superior temporal **(C)**. Superior temporal sulcus: superior temporal **(D1)**, middle temporal **(D2)**. Intra-parietal sulcus: superior parietal **(E1)**, inferior parietal **(E2)**.

Five sulci in each hemisphere were chosen for analysis including (Figure 2): (A) superior frontal sulcus, (B) central sulcus, (C) lateral sulcus, (D) superior temporal sulcus, and (E) intra-parietal sulcus. These were chosen because they are present in all individuals; they are large and relatively easy to identify with precision (Liu et al., 2010); and they are located in or adjoining each cerebral lobe, and thus may better reflect the volumetric changes occurring in these regions.

#### GM and WM ROIs

GM and WM volumetric estimates were produced for 34 regions in each hemisphere (Liu et al., 2013b). The regions adjacent to the five selected sulci were chosen as local factors (LF) to index local impact on sulcal changes. They included: pre-central, post-central, superior frontal, middle frontal, superior temporal, middle temporal, superior parietal and inferior parietal (Figure 2). In addition, total cortical GM and WM volumes were selected as global factors (GF). Total cortical GM was chosen rather than whole brain GM, because the latter includes subcortical and cerebellar GM which are thought to have less impact on sulcal morphology (Liu et al., 2013b).

### Statistical Analyses

All statistical analyses were conducted using IBM SPSS 20.0. The associations between sulcal characteristics and age were investigated with multiple linear regression analyses controlling for sex, intracranial volume (ICV), education and APOE ε4 genotype. The effect of age was investigated by contrasting MA and OA and by testing the effect of age within group (AgeC). For this purpose, two variables were computed, one reflecting whether participants were in the MA or OA group, and another reflecting age variance within group (AgeC). For AgeC, the age of each participant was centered on the mean of their group by subtracting the rounded minimum age of their age group (45 and 65) from their age.

To test whether LF or GF were more strongly associated with sulcal characteristics, as well as to compare the sensitivity of sulcal width and depth to local brain volumetric differences, multiple regression analyses were used and differences in ΔR^2^ were tested between models. We applied Bonferroni corrections and considered *p* < 0.01 as statistically significant given five sulci were investigated.

## Results

### Demographics

Demographics are shown in Table 1. MA participants did not differ significantly on age (*p* = 0.180), sex (*p* = 0.708) and years of education (*p* = 0.752) from the whole 40+ PATH cohort. Likewise, OA participants did not differ significantly on age (*p* = 0.473), sex (*p* = 0.077), and years of education (*p* = 0.912) from the whole 60+ PATH cohort. MA and OA participants differed significantly from each other on age, sex, years of education, Goldberg depression score, smoking, diabetes and hypertension (*p* < 0.05, see Table 1).

**Table 1:** Demographic characteristics.

Characteristics	MA (*N* = 403)	OA (*N* = 390)	*t* or *χ*^2*^	*p*
Age, years (SD)	47.20 (1.37)	67.04 (1.42)	−199.6	<0.001
Range	44–50	64–70	-	-
Male, *N* (%)	186 (46.15)	220 (56.41)	8.149	0.004
Caucasian, *N* (%)	385 (95.53)	368 (94.36)	0.559	0.518
Education, years (SD)	15.12 (4.74)	14.16 (2.62)	4.035	<0.001
BMI, score (SD)	27.16 (4.80)	26.59 (4.24)	1.860	0.064
Goldberg depression score (SD)	2.25 (2.25)	2.20 (7.20)	3.735	<0.001
Smoking (ever), N (%)	193 (47.89)	168 (43.08)	1.752	0.199
Diabetes, *N* (%)	10 (2.48)	40 (10.26)	20.20	<0.001
Hypertension, *N* (%)	114 (28.29)	258 (66.15)	113.5	<0.001
MMSE, score (SD)	-	29.53 (3.66)	-	-

*Comparing MA and OA, t-test for continuous variables and Chi-squared test for categorical variables.

### Age Differences in Sulcal Characteristics

#### Sulcal Width

**Figure 3:**
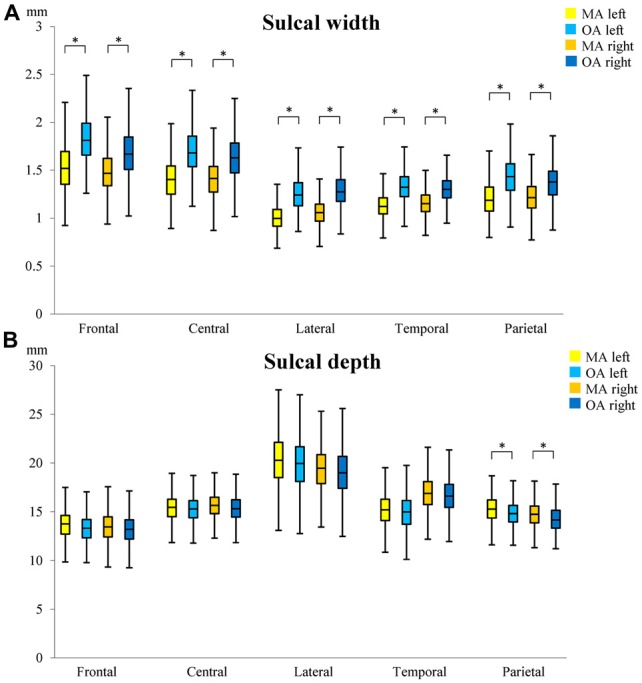
Differences in sulcal width **(A)** and depth **(B)** between middle-age (MA) and old-age (OA). The median, 1st and 3rd quartile range of sulcal widths and depths in MA and OA are shown. Whiskers show minimum and maximum value. *Indicates significant (*p* < 0.01) age group differences in sulcal width/depth. Superior frontal sulcus (frontal); central sulcus (central), lateral sulcus (lateral); superior temporal sulcus (temporal); intra-parietal sulcus (parietal).

The mean sulcal width of sulci investigated was 1.27 mm (SD = 0.17 mm) in MA and 1.49 mm (SD = 0.20 mm) in OA representing a 17.3% difference between age groups 20 years apart (0.87%/year, *p* < 0.001; see Supplementary Table S1 for details). In MA, the sulcal widths of all 10 sulci were significantly narrower than in OA (Figure 3A). The left superior frontal sulcus showed the largest sulcal difference (0.341 mm; 22.11%) and the right intra-parietal sulcus the lowest (0.154 mm; 13.25%). In general, an anterior to posterior topographical gradient in sulcal width was observed such that sulcal widths became narrower from the frontal lobe to the occipital lobe in both age groups. No significant association between sulcal width and age (AgeC) was detected within the 4-year age bands of the two age groups. In addition, a lack of a significant age by age group interaction (AgeC × AgeG) indicated that the rate of sulcal widening with age did not differ between MA and OA (see Supplementary Table S2).

#### Sulcal Depth

The mean depth of sulci investigated was 15.98 mm (SD = 2.12 mm) in MA and 15.67 mm (SD = 2.07 mm) in OA representing a 1.93% difference between age groups 20 years apart (0.1%/year). The sulcal depths of the left and right intra-parietal sulci were significantly deeper in MA than in OA (*p* < 0.01; Figure 3B). The right intra-parietal sulcus showed a larger sulcal difference (0.442 mm; 3.00%) between groups than the left. No significant association between sulcal depth and age (AgeC) was detected within age group. The age by age group interaction (AgeC × AgeG) showed that, in the left intra-parietal sulcus, there was a significant difference in the rate of annual change in sulcal depth (MA: 0.086 mm/year; OA: −0.097 mm/year; see Supplementary Table S3).

#### Sex Differences in Sulcal Characteristics

**Figure 4:**
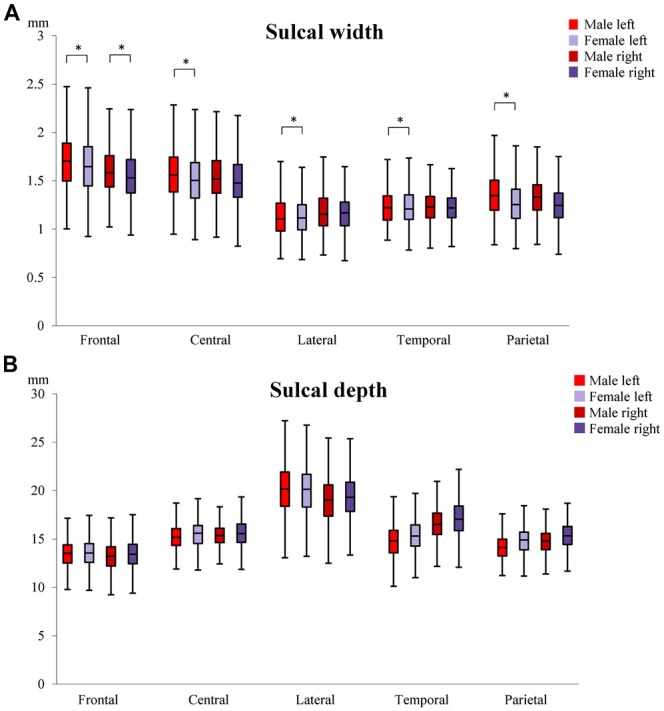
Differences in sulcal width **(A)** and depth **(B)** between males and females. The median, 1st and 3rd quartile range of sulcal widths and depths in MA and OA are shown. Whiskers show minimum and maximum value. *Indicates significant (*p* < 0.01) sex by age group differences in sulcal width/depth. Superior frontal sulcus (frontal); central sulcus (central), lateral sulcus (lateral); superior temporal sulcus (temporal); intra-parietal sulcus (parietal).

To determine whether sulcal characteristics differed between males and females in MA and OA, sex by age group interactions (Sex × AgeG) were tested. For sulcal width, significant differences were observed in the left lateral sulcus (males = 0.278 mm, females = 0.205 mm), left intra-parietal sulcus (males = 0.251 mm, females = 0.171 mm), left central sulcus (males = 0.333 mm, females = 0.250 mm), left (males = 0.341 mm, females = 0.251 mm) and right (males = 0.226 mm, females = 0.134 mm) superior frontal sulci (Figure 4A). In general, the change in sulcal width was significantly higher in male than female. No significant difference was detected in sulcal depth (Figure 4B; Supplementary Tables S2, S3).

#### Laterality Differences in Sulcal Characteristics

The average sulcal width of all 10 sulci across both age groups was 1.39 mm (SD 0.24) in the left hemisphere and 1.37 mm (SD 0.18) in the right. The average sulcal depth of all 10 sulci across both age groups was 15.80 mm (SD 2.19) in the left hemisphere and 15.85 mm (SD 2.00) in the right. In order to test whether the sulcal measures were different between the two hemispheres across both age groups, left and right sulcal characteristics were compared. For sulcal width, a significant difference between the left and right hemispheres was observed in the intra-parietal sulcus (the confidence intervals did not overlap, see Supplementary Table S2). For sulcal depth, no significant differences between the left and right hemispheres were found.

### Association Between Sulcal Characteristics and Local and Global Factors

**Figure 5:**
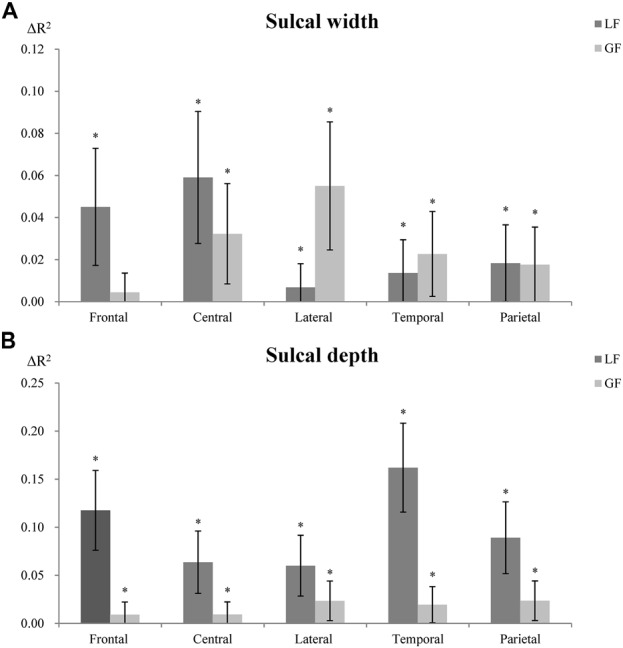
Contribution of LF and GF to sulcal width **(A)** and depth **(B)**. Local factors (LF); global factors (GF). The R-squared changes (ΔR^2^) indicate changes of explanatory power between models with and without LF or GF (proportion of variability of the sulcal measures explained by LF or GF). *Indicates significant association (*p* < 0.01) between LF or GF and sulcal width or depth. Error bar shows 95% confidence interval, which indicates significant differences (95% confidence interval not overlapping) between LF and GF in explaining sulcal width/depth.

LF were more predictive of sulcal width in the superior frontal sulcus, while GF were more predictive in the lateral sulcus (Figure 5A). For sulcal depth, LF were generally more predictive in most sulci (Figure 5B, Supplementary Tables S4–S6). LF and GF were significantly correlated, with *r* ranging from 0.872 to 0.944.

### Sensitivity of Sulcal Width and Depth to Local Brain Volume Differences

**Figure 6:**
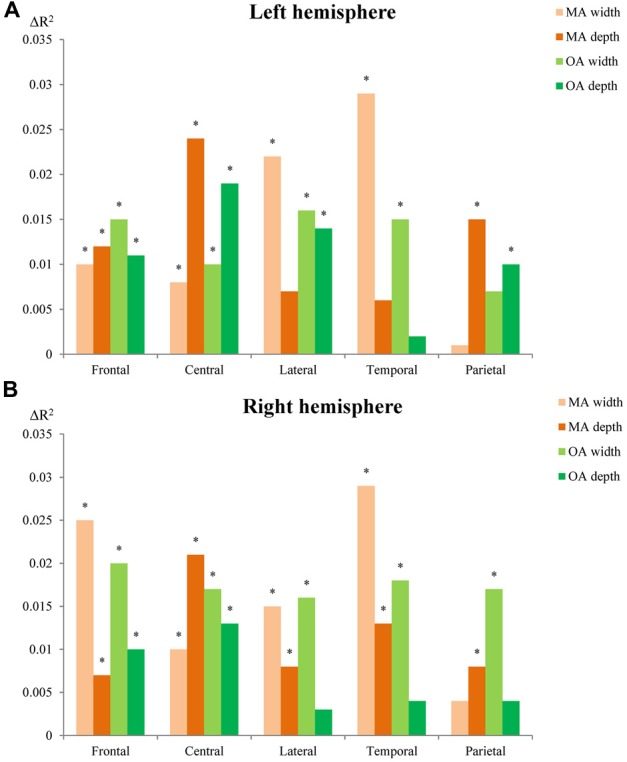
Sensitivity of sulcal width and depth to local brain volumetric differences in the left **(A)** and right **(B)** hemispheres. MA, Middle age; OA, old age. The R-squared changes (ΔR^2^) indicate changes of explanatory power between models with and without sulcal width or depth (proportion of variability in the local gyral volumes explained by sulcal width or depth). *Indicates significant association (*p* < 0.01) between local gyral volumes and sulcal width or depth.

To determine whether sulcal width or depth was more predictive of local gyral volumes in the left (Figure 6A) or the right (Figure 6B) hemispheres, the two measures were entered together in regression analyses (Supplementary Table S7). They revealed that sulcal width was generally more strongly associated with local brain volume differences in the superior frontal sulcus, lateral sulcus and superior temporal sulcus, while sulcal depth was generally more strongly associated with local brain volume in the central sulcus and intra-parietal sulcus suggesting a ventro-anterior to dorso-posterior gradient. For specific differences, sulcal width was significantly associated with local gyral volume at right intra-parietal sulcus but not left intra-parietal sulcus in either OA or MA. Sulcal depth was significantly associated with local gyral volume at left lateral sulcus in OA, at right temporal sulcus in MA and at left intra-parietal sulcus in both MA and OA and right intra-parietal sulcus in MA.

## Discussion

This study produced four main findings. In this large population-based cohort: (1) sulci were wider in older participants in all five sulci investigated; (2) sulcal depth was significantly shallower in older participants in the intra-parietal sulcus; (3) LF were generally more predictive of sulcal morphology than GF; and (4) sulcal width was generally more strongly associated with local brain volumetric differences.

### Age, Sex and Laterality

As predicted and generally consistent with the literature the 10 sulci investigated were wider in older participants (Kochunov et al., 2005; Liu et al., 2010). However, for sulcal depth, unlike previous findings, results showed that although depths of the 10 sulci were generally shallower in older participants, only the intra-parietal sulcus was significantly shallower. The reason for this discrepancy may have been due to cohort differences. In Kochunov’s study, participants’ age spanned a very wide age range of 20–82 years and therefore might have been more likely to present larger sulcal differences.

The largest difference in sulcal width between the two age groups (12.85%) was found in the left superior frontal sulcus. This pattern has been previously reported (Kochunov et al., 2005) and is consistent with anatomical studies of aging that show accelerated GM atrophy in the superior frontal region (Wu et al., 1996; Xu et al., 2000; Good et al., 2001; Raz and Rodrigue, 2006; Smith et al., 2007; Fjell et al., 2009). It has been suggested that frontal regions mature later during development (Gogtay et al., 2004), and thus may be more vulnerable to aging (Fjell et al., 2014). The reason for this effect has not been elucidated but may be due to a difference in cortical architecture (Fjell et al., 2014). Moreover, the age-related widening was found to generally follow an anterior to posterior gradient. This is likely to be related to the fact that the temporal and occipital lobes experience smaller volumetric decline with age compared to the frontal and parietal lobes (Resnick et al., 2003).

Analyses of sex differences showed significant effects, which varied between age groups such that males had greater sulcal width than females in OA (right lateral, left parietal, and left central sulci as well as in the left and right superior frontal sulci) but not in MA. The opposite effect was found for the left lateral sulcus. Importantly, these differences were detected after controlling for ICV and therefore cannot be attributed to known differences in head size between sexes. A previous study also found age-related sulcal difference in men and women, such as different sulcal width in males than females in the superior temporal (males: 0.83 mm/decade; females: 0.58 mm/decade), collateral (males: 0.82 mm/decade; females: 0.54 mm/decade), and cingulate sulci (males: 0.88 mm/decade; females: 0.66 mm/decade; Kochunov et al., 2005). It is likely that these differences are driven by a number of factors. In addition to clearly demonstrated variation in some physiological and hormonal processes, men and women have different exposure to demonstrated risk factors for neurodegeneration (Cherbuin et al., 2015), vary in their genetic predisposition to neurodegenerative conditions such as Alzheimer’s disease, and make different lifestyle choices which relate to cerebral health (e.g., smoking, diet, exercise, etc.). These differences are likely to contribute to the documented faster cortical GM atrophy in men (Curiati et al., 2009). Moreover, men have also been found to have less cortical gyrification than women which may also contribute to variation in sulcal widening (Luders et al., 2004).

Significant sulcal width differences in the left and right hemispheres were observed in the intra-parietal sulci and are consistent with lateralization of attention processes and reported structural asymmetries in the parietal cortex (Jeong and Xu, 2016). However, most sulci showed no significant differences in laterality. This is surprising because laterality differences might have been expected in the central sulcus due to handedness (Mangin et al., 2004a), or in the medial frontal lobe and superior temporal gyrus due to their implication in language processing which is known to be lateralized to the left hemisphere in most individuals (Foundas et al., 1994; Holland et al., 2007; Vannest et al., 2009).

### Influence of Local and Global Factors on Sulcal Characteristics

An important question to resolve in order to assess sulcal characteristics as indexes of brain atrophy is whether they are more reflective of regional brain changes proximal to a specific gyrus or whether they integrate more global changes distributed more diffusely across the whole brain. One issue is that local and global brain volumes are highly correlated, resulting in overlap in explanatory power. Nevertheless, results from this study have identified significant difference in associations between LF/GF and sulcal characteristics in some sulci. Our findings show that LF were more predictive of sulcal width in the superior frontal sulcus, while GF were more predictive of sulcal width in the lateral sulcus. Further, LF were more strongly associated with sulcal depth across all gyri investigated. These results are not completely consistent with our working hypotheses and previous studies which found that local GM was typically more strongly associated with sulcal width than global GM (Liu et al., 2013b). This discrepancy may be explained by the fact that Liu and colleagues did not consider the contribution of WM. Since both local GM and WM contribute to sulcal variability (Im et al., 2008) our findings might reflect different local and global influences of WM on sulcal characteristics in different brain regions.

Although it has been suggested that sulcal changes adjoining certain gyri may indicate loss in function supported by these gyri (Rettmann et al., 2006), our findings suggest a more complex story. Indeed, we found that sulcal width was more strongly associated with the volumes of local brain regions adjoining the superior frontal sulcus, but that global brain volume was more strongly associated with the sulcal width of the lateral sulcus. Moreover, local and global volumes contributed similarly to the width of the central, temporal and parietal sulci. In contrast, sulcal depth of all sulci was consistently more strongly associated with the volumes of adjacent gyri than global volumes. Thus, although we cannot investigate this question in the present study, it would seem that age-related differences in sulcal width and depth may be generally more reflective of functional changes related to local gyri, although there may be a number of exceptions in relation to the width of certain gyri.

### Sulcal Width and Depth and Local Brain Volumes

In addition to clarifying how LF or GF contribute to sulcal morphology, an important outstanding question is whether sulcal width or depth is more strongly associated with local gyral structure, when both measures are considered together. We found that the width of the superior frontal, lateral and superior temporal sulci were more predictive of the adjoining gyral volumes. In contrast, sulcal depth of the central and intra-parietal sulci was more predictive of local volumes.

This study had a number of limitations but also several strengths. The cross-sectional design used does not allow for causal inferences and longitudinal investigations are needed to confirm these findings. Moreover, the narrow-age cohort design used within each age group and the large age difference between age groups did not allow us to investigate whether age effects are progressive between MA and OA or whether they follow a non-linear trend. However, this design characteristic is also a strength as it allows for more precise measures of sulcal characteristics within the selected age ranges.

## Conclusion

Sulci were on average 17.3% wider in OA compared to MA, with the left superior frontal sulcus showing the largest difference (22.11%). The left and right intra-parietal sulci were also significantly shallower in OA than in MA. Importantly, we found that the volume of gray and WM adjacent to the superior frontal sulcus (LF) was highly predictive of the variability in width of this sulcus, while whole brain gray and white matter volumes (GF) were more predictive of sulcal width in the lateral sulcus. These results suggest that sulcal characteristics can provide unique information about changes in local and global brain structure in aging.

## Author Contributions

KJ and TZ contributed to the design of the study, provided methodological input and theoretical expertise, conducted the statistical analyses and all aspects of manuscript preparation and submission. MS provided methodological input and theoretical expertise, contributed to statistical analyses and contributed to writing and editing of the manuscript. PS contributed to the design of the study, provided methodological input and theoretical expertise and contributed to writing and editing of the manuscript. NC contributed to the design of the study, provided methodological input and theoretical expertise, contributed to statistical analyses and contributed to writing and editing of the manuscript.

## Conflict of Interest Statement

The authors declare that the research was conducted in the absence of any commercial or financial relationships that could be construed as a potential conflict of interest.

## Footnotes

^1^http://freesurfer.net/

^2^http://brainvisa.info/

## Abbreviations

AgeC: age within group
AgeG: age group (middle age or old age)
GM: gray matter
ICV: intracranial volume
MA: middle age
OA: old age
PATH: Personality and Total Health Through Life
ROI: region of interest
WM: white matter.
