# Supplementary material for: Relationship Between Sulcal Characteristics and Brain Aging
Source: Front Aging Neurosci. 2018 Nov 12;10:339. doi: 10.3389/fnagi.2018.00339 (PMC6240579; doi:10.3389/fnagi.2018.00339)
Supplement: Supplementary file 1 [file Data_Sheet_1.docx]

# Supplementary Tables

**Table 1. Mean and standard deviations of sulcal measures of different age and sex groups.** Sulci: lateral sulcus (lateral); intra-parietal sulcus (parietal); central sulcus (central); superior frontal sulcus (frontal); superior temporal sulcus (temporal)

| Sulcal width | MA | | OA | | Male | | Female | |
| --- | --- | --- | --- | --- | --- | --- | --- | --- |
|  | Width | SD | Width | SD | Width | SD | Width | SD |
| Left lateral | 1.02 | 0.18 | 1.26 | 0.19 | 1.13 | 0.16 | 1.15 | 0.19 |
| Right lateral | 1.06 | 0.17 | 1.29 | 0.19 | 1.17 | 0.16 | 1.19 | 0.17 |
| Left parietal | 1.22 | 0.27 | 1.43 | 0.22 | 1.35 | 0.18 | 1.30 | 0.26 |
| Right parietal | 1.23 | 0.23 | 1.37 | 0.20 | 1.33 | 0.17 | 1.27 | 0.23 |
| Left central | 1.41 | 0.27 | 1.70 | 0.26 | 1.57 | 0.22 | 1.54 | 0.28 |
| Right central | 1.41 | 0.23 | 1.64 | 0.25 | 1.54 | 0.21 | 1.51 | 0.24 |
| Left frontal | 1.54 | 0.32 | 1.84 | 0.28 | 1.70 | 0.26 | 1.68 | 0.30 |
| Right frontal | 1.49 | 0.30 | 1.69 | 0.28 | 1.60 | 0.24 | 1.58 | 0.31 |
| Left temporal | 1.14 | 0.25 | 1.33 | 0.16 | 1.22 | 0.14 | 1.25 | 0.23 |
| Right temporal | 1.16 | 0.17 | 1.30 | 0.15 | 1.23 | 0.14 | 1.24 | 0.16 |
|  |  |  |  |  |  |  |  |  |
|  |  |  |  |  |  |  |  |  |
| Sulcal depth | MA | | OA | | Male | | Female | |
|  | Depth | SD | Depth | SD | Depth | SD | Depth | SD |
| Left lateral | 20.13 | 3.01 | 19.73 | 3.04 | 19.77 | 2.91 | 19.94 | 2.84 |
| Right lateral | 19.23 | 2.66 | 18.82 | 2.74 | 18.72 | 2.59 | 19.33 | 2.63 |
| Left parietal | 15.17 | 1.63 | 14.83 | 1.46 | 14.67 | 1.31 | 15.29 | 1.39 |
| Right parietal | 14.69 | 1.64 | 14.23 | 1.44 | 13.99 | 1.23 | 14.81 | 1.42 |
| Left central | 15.40 | 1.58 | 15.24 | 1.51 | 15.15 | 1.27 | 15.49 | 1.44 |
| Right central | 15.55 | 1.59 | 15.33 | 1.46 | 15.26 | 1.23 | 15.57 | 1.41 |
| Left frontal | 13.68 | 1.62 | 13.37 | 1.60 | 13.39 | 1.43 | 13.58 | 1.52 |
| Right frontal | 13.42 | 1.74 | 13.19 | 1.62 | 13.15 | 1.58 | 13.41 | 1.55 |
| Left temporal | 15.12 | 1.89 | 14.98 | 1.89 | 14.78 | 1.70 | 15.37 | 1.78 |
| Right temporal | 16.92 | 1.89 | 16.63 | 1.88 | 16.46 | 1.54 | 17.09 | 1.88 |

All subjects

|  |  |  |  |  |  |  |  |  |
| --- | --- | --- | --- | --- | --- | --- | --- | --- |
|  | Left width | SD | Right width | SD | Left depth | SD | Right depth | SD |
| Lateral | 1.14 | 0.18 | 1.18 | 0.17 | 19.98 | 2.90 | 19.12 | 2.61 |
| Parietal | 1.33 | 0.23 | 1.31 | 0.21 | 15.04 | 1.37 | 14.49 | 1.37 |
| Central | 1.56 | 0.26 | 1.53 | 0.23 | 15.34 | 1.37 | 15.47 | 1.33 |
| Frontal | 1.69 | 0.29 | 1.59 | 0.28 | 13.55 | 1.48 | 13.34 | 1.58 |
| Temporal | 1.24 | 0.20 | 1.23 | 0.15 | 15.09 | 1.76 | 16.82 | 1.75 |

**Table 2. Multiple regression analysis of the associations between age and sulcal width, controlling for Sex, ICV, Education and APOE.** The table includes age group (AgeG), age centred (AgeC), age interaction (AgeC x AgeG) and Sex interaction (Sex x AgeG). AgeG: age group; AgeC: age centred on the mean of their group by subtracting the rounded minimum age of their age group (45 and 65) from their age. The significance level is set at p < 0.01. Significant results are shown in bold. Sulci: lateral sulcus (lateral); intra-parietal sulcus (parietal); central sulcus (central); superior frontal sulcus (frontal); superior temporal sulcus (temporal)

| **Sulcal Width** | | Coefficient | std.error | P value | Confidence interval | |
| --- | --- | --- | --- | --- | --- | --- |
|  |  |  |  |  | Lower Bound | Upper Bound |
| **Left lateral** | AgeG | **0.278** | **0.019** | **0** | **0.242** | **0.315** |
|  | AgeC | 0.005 | 0.007 | 0.455 | -0.008 | 0.018 |
|  | AgeC x AgeG | 0.007 | 0.009 | 0.442 | -0.011 | 0.026 |
|  | Sex x AgeG | **-0.073** | **0.027** | **0.006** | **-0.125** | **-0.021** |
|  |  |  |  |  |  |  |
| **Right lateral** | AgeG | **0.239** | **0.018** | **0** | **0.204** | **0.274** |
|  | AgeC | 0.005 | 0.006 | 0.441 | -0.008 | 0.018 |
|  | AgeC x AgeG | 0.014 | 0.009 | 0.135 | -0.004 | 0.031 |
|  | Sex x AgeG | -0.025 | 0.025 | 0.318 | -0.075 | 0.025 |
|  |  |  |  |  |  |  |
| **Left parietal** | AgeG | **0.251** | **0.024** | **0** | **0.204** | **0.298** |
|  | AgeC | 0.01 | 0.009 | 0.246 | -0.007 | 0.028 |
|  | AgeC x AgeG | 0.008 | 0.012 | 0.511 | -0.016 | 0.032 |
|  | Sex x AgeG | **-0.08** | **0.035** | **0.022** | **-0.148** | **-0.011** |
|  |  |  |  |  |  |  |
| **Right parietal** | AgeG | **0.154** | **0.021** | **0** | **0.112** | **0.196** |
|  | AgeC | 0.009 | 0.008 | 0.263 | -0.007 | 0.024 |
|  | AgeC x AgeG | 0.003 | 0.011 | 0.779 | -0.018 | 0.025 |
|  | Sex x AgeG | -0.047 | 0.031 | 0.127 | -0.108 | 0.013 |
|  |  |  |  |  |  |  |
| **Left central** | AgeG | **0.333** | **0.026** | **0** | **0.281** | **0.385** |
|  | AgeC | 0.018 | 0.01 | 0.06 | -0.001 | 0.037 |
|  | AgeC x AgeG | -0.001 | 0.014 | 0.918 | -0.028 | 0.025 |
|  | Sex x AgeG | **-0.083** | **0.038** | **0.029** | **-0.158** | **-0.008** |
|  |  |  |  |  |  |  |
| **Right central** | AgeG | **0.237** | **0.024** | **0** | **0.19** | **0.284** |
|  | AgeC | 0.016 | 0.009 | 0.07 | -0.001 | 0.033 |
|  | AgeC x AgeG | -0.005 | 0.012 | 0.684 | -0.029 | 0.019 |
|  | Sex x AgeG | -0.028 | 0.034 | 0.412 | -0.096 | 0.039 |
|  |  |  |  |  |  |  |
| **Left frontal** | AgeG | **0.341** | **0.03** | **0** | **0.282** | **0.401** |
|  | AgeC | 0.007 | 0.011 | 0.506 | -0.014 | 0.029 |
|  | AgeC x AgeG | 0.011 | 0.015 | 0.477 | -0.019 | 0.041 |
|  | Sex x AgeG | **-0.09** | **0.043** | **0.039** | **-0.175** | **-0.004** |
|  |  |  |  |  |  |  |
| **Right frontal** | AgeG | **0.226** | **0.029** | **0** | **0.169** | **0.284** |
|  | AgeC | -0.002 | 0.011 | 0.815 | -0.023 | 0.018 |
|  | AgeC x AgeG | 0.013 | 0.015 | 0.392 | -0.017 | 0.042 |
|  | Sex x AgeG | **-0.092** | **0.042** | **0.029** | **-0.174** | **-0.01** |
|  |  |  |  |  |  |  |
| **Left temporal** | AgeG | **0.216** | **0.021** | **0** | **0.175** | **0.257** |
|  | AgeC | 0.003 | 0.008 | 0.646 | -0.011 | 0.018 |
|  | AgeC x AgeG | 0.009 | 0.011 | 0.371 | -0.011 | 0.03 |
|  | Sex x AgeG | -0.058 | 0.03 | 0.05 | -0.117 | 0 |
|  |  |  |  |  |  |  |
| **Right temporal** | AgeG | **0.161** | **0.016** | **0** | **0.129** | **0.193** |
|  | AgeC | 0.004 | 0.006 | 0.482 | -0.007 | 0.016 |
|  | AgeC x AgeG | 0.005 | 0.008 | 0.571 | -0.012 | 0.021 |
|  | Sex x AgeG | -0.038 | 0.023 | 0.101 | -0.084 | 0.007 |

**Table 3. Multiple regression analysis of the associations between age and sulcal depth, controlling for Sex, ICV, Education and APOE.** The table includes age group (AgeG), age centred (AgeC), age interaction (AgeC x AgeG) and Sex interaction (Sex x AgeG). AgeG: age group; AgeC: age centred on the mean of their group by subtracting the rounded minimum age of their age group (45 and 65) from their age. The significance level is set at p < 0.01. Significant results are shown in bold. Sulci: lateral sulcus (lateral); intra-parietal sulcus (parietal); central sulcus (central); superior frontal sulcus (frontal); superior temporal sulcus (temporal)

| **Sulcal Depth** | | Coefficient | std.error | P value | Confidence interval | |
| --- | --- | --- | --- | --- | --- | --- |
|  |  |  |  |  | Lower Bound | Upper Bound |
| **Left lateral** | AgeG | -0.521 | 0.298 | 0.081 | -1.105 | 0.063 |
|  | AgeC | 0.155 | 0.109 | 0.153 | -0.058 | 0.368 |
|  | AgeC x AgeG | -0.138 | 0.152 | 0.365 | -0.436 | 0.161 |
|  | Sex x AgeG | 0.363 | 0.428 | 0.396 | -0.477 | 1.202 |
|  |  |  |  |  |  |  |
| **Right lateral** | AgeG | -0.328 | 0.269 | 0.223 | -0.855 | 0.2 |
|  | AgeC | -0.065 | 0.098 | 0.507 | -0.258 | 0.127 |
|  | AgeC x AgeG | 0.175 | 0.137 | 0.201 | -0.094 | 0.445 |
|  | Sex x AgeG | -0.177 | 0.386 | 0.647 | -0.935 | 0.581 |
|  |  |  |  |  |  |  |
| **Left parietal** | AgeG | **-0.339** | **0.137** | **0.010** | **-0.608** | **-0.07** |
|  | AgeC | 0.086 | 0.05 | 0.088 | -0.013 | 0.184 |
|  | AgeC x AgeG | **-0.183** | **0.07** | **0.009** | **-0.32** | **-0.046** |
|  | Sex x AgeG | 0.075 | 0.197 | 0.705 | -0.312 | 0.461 |
|  |  |  |  |  |  |  |
| **Right parietal** | AgeG | **-0.432** | **0.134** | **0.001** | **-0.694** | **-0.169** |
|  | AgeC | 0.018 | 0.049 | 0.719 | -0.078 | 0.113 |
|  | AgeC x AgeG | -0.094 | 0.068 | 0.168 | -0.228 | 0.04 |
|  | Sex x AgeG | 0.116 | 0.192 | 0.548 | -0.262 | 0.493 |
|  |  |  |  |  |  |  |
| **Left central** | AgeG | -0.093 | 0.137 | 0.494 | -0.362 | 0.175 |
|  | AgeC | 0.022 | 0.05 | 0.658 | -0.076 | 0.12 |
|  | AgeC x AgeG | -0.071 | 0.07 | 0.307 | -0.208 | 0.066 |
|  | Sex x AgeG | -0.032 | 0.196 | 0.871 | -0.417 | 0.354 |
|  |  |  |  |  |  |  |
| **Right central** | AgeG | -0.237 | 0.132 | 0.072 | -0.496 | 0.021 |
|  | AgeC | 0.052 | 0.048 | 0.282 | -0.043 | 0.146 |
|  | AgeC x AgeG | -0.071 | 0.067 | 0.292 | -0.203 | 0.061 |
|  | Sex x AgeG | 0.107 | 0.189 | 0.571 | -0.264 | 0.478 |
|  |  |  |  |  |  |  |
| **Left frontal** | AgeG | -0.276 | 0.153 | 0.072 | -0.576 | 0.024 |
|  | AgeC | 0.001 | 0.056 | 0.991 | -0.109 | 0.11 |
|  | AgeC x AgeG | 0.015 | 0.078 | 0.846 | -0.138 | 0.168 |
|  | Sex x AgeG | -0.06 | 0.22 | 0.783 | -0.492 | 0.371 |
|  |  |  |  |  |  |  |
| **Right frontal** | AgeG | -0.297 | 0.162 | 0.067 | -0.615 | 0.021 |
|  | AgeC | 0.066 | 0.059 | 0.268 | -0.051 | 0.182 |
|  | AgeC x AgeG | -0.095 | 0.083 | 0.249 | -0.258 | 0.067 |
|  | Sex x AgeG | 0.101 | 0.233 | 0.665 | -0.356 | 0.558 |
|  |  |  |  |  |  |  |
| **Left temporal** | AgeG | -0.092 | 0.178 | 0.604 | -0.441 | 0.257 |
|  | AgeC | -0.1 | 0.065 | 0.124 | -0.227 | 0.027 |
|  | AgeC x AgeG | 0.024 | 0.091 | 0.792 | -0.154 | 0.202 |
|  | Sex x AgeG | -0.142 | 0.255 | 0.577 | -0.644 | 0.359 |
|  |  |  |  |  |  |  |
| **Right temporal** | AgeG | -0.146 | 0.176 | 0.406 | -0.492 | 0.199 |
|  | AgeC | -0.001 | 0.064 | 0.987 | -0.127 | 0.125 |
|  | AgeC x AgeG | 0.013 | 0.09 | 0.884 | -0.163 | 0.189 |
|  | Sex x AgeG | -0.218 | 0.253 | 0.388 | -0.714 | 0.278 |

## Table 4. Association between sulcal measures and local and global factors.

Positive associations are shown in yellow and negative associations are shown in blue. Significant results are shown in green. The significance level was set at p < 0.01 as previously mentioned. ROI GM refer to grey matter volumes of ROIs, while ROI WM refer to white matter volumes of ROIs. Cortical GM refers to cortical grey matter volume and Cortical WM refers to cortical white matter volume. Age groups: middle age (40); old age (60).

**Sulcal width**

1. Superior frontal sulcus

Left hemisphere

| Model | | Unstandardized Coefficients | | Sig. |
| --- | --- | --- | --- | --- |
|  |  | B | Std. Error |  |
| 40 | (Constant) | 1.566 | .565 | .006 |
|  | Superior frontal GM | -5.043E-05 | .000 | .005 |
|  | Middle frontal GM | 3.178E-05 | .000 | .125 |
|  | Superior frontal WM | 2.867E-06 | .000 | .880 |
|  | Middle frontal WM | -5.844E-05 | .000 | .008 |
|  | Cortical GM | -5.750E-07 | .000 | .605 |
|  | Cortical WM | 1.649E-06 | .000 | .069 |
|  | Age | .003 | .011 | .757 |
|  | Sex | -.004 | .038 | .922 |
|  | ICV | 5.148E-07 | .000 | .005 |
| 60 | (Constant) | 2.474 | .653 | .000 |
|  | Superior frontal GM | -2.834E-05 | .000 | .095 |
|  | Middle frontal GM | 2.342E-05 | .000 | .163 |
|  | Superior frontal WM | -9.129E-06 | .000 | .597 |
|  | Middle frontal WM | -2.257E-05 | .000 | .220 |
|  | Cortical GM | -2.783E-06 | .000 | .024 |
|  | Cortical WM | -1.194E-06 | .000 | .207 |
|  | Age | -.001 | .009 | .876 |
|  | Sex | -.117 | .033 | .000 |
|  | ICV | 1.216E-06 | .000 | .000 |
| 40+60 | (Constant) | 1.903 | .137 | .000 |
|  | Superior frontal GM | -3.705E-05 | .000 | .003 |
|  | Middle frontal GM | 2.236E-05 | .000 | .090 |
|  | Superior frontal WM | -6.366E-06 | .000 | .620 |
|  | Middle frontal WM | -3.926E-05 | .000 | .006 |
|  | Cortical GM | -1.086E-06 | .000 | .182 |
|  | Cortical WM | 3.780E-07 | .000 | .564 |
|  | AgeG | .203 | .025 | .000 |
|  | AgeC | .003 | .007 | .652 |
|  | Sex | -.056 | .025 | .026 |
|  | ICV | 7.619E-07 | .000 | .000 |

Right hemisphere

| Model | | Unstandardized Coefficients | | Sig. |
| --- | --- | --- | --- | --- |
|  |  | B | Std. Error |  |
| 40 | (Constant) | 1.745 | .525 | .001 |
|  | Superior frontal GM | -4.907E-05 | .000 | .003 |
|  | Middle frontal GM | 4.278E-05 | .000 | .011 |
|  | Superior frontal WM | -3.066E-06 | .000 | .859 |
|  | Middle frontal WM | -5.586E-05 | .000 | .001 |
|  | Cortical GM | -1.653E-06 | .000 | .124 |
|  | Cortical WM | 1.781E-06 | .000 | .024 |
|  | Age | .000 | .010 | .967 |
|  | Sex | -.011 | .035 | .752 |
|  | ICV | 5.631E-07 | .000 | .001 |
| 60 | (Constant) | 2.375 | .650 | .000 |
|  | Superior frontal GM | -5.440E-05 | .000 | .000 |
|  | Middle frontal GM | 3.891E-05 | .000 | .022 |
|  | Superior frontal WM | 1.076E-05 | .000 | .508 |
|  | Middle frontal WM | -3.903E-05 | .000 | .036 |
|  | Cortical GM | -1.806E-06 | .000 | .154 |
|  | Cortical WM | -1.509E-06 | .000 | .072 |
|  | Age | -.008 | .009 | .402 |
|  | Sex | -.079 | .033 | .015 |
|  | ICV | 1.348E-06 | .000 | .000 |
| 40+60 | (Constant) | 1.755 | .131 | .000 |
|  | Superior frontal GM | -4.974E-05 | .000 | .000 |
|  | Middle frontal GM | 3.901E-05 | .000 | .001 |
|  | Superior frontal WM | 1.314E-06 | .000 | .912 |
|  | Middle frontal WM | -4.671E-05 | .000 | .000 |
|  | Cortical GM | -1.441E-06 | .000 | .075 |
|  | Cortical WM | 2.604E-07 | .000 | .650 |
|  | AgeG | .089 | .023 | .000 |
|  | AgeC | -.001 | .007 | .892 |
|  | Sex | -.046 | .024 | .057 |
|  | ICV | 8.800E-07 | .000 | .000 |

1. Central sulcus

Left hemisphere

| Model | | Unstandardized Coefficients | | | | Sig. | |  |
| --- | --- | --- | --- | --- | --- | --- | --- | --- |
|  |  | B | | Std. Error | |  |  |  |
| 40 | (Constant) | .785 | | .461 | | .089 | |  |
|  | Postcentral GM | 3.443E-05 | | .000 | | .148 | |  |
|  | Precentral GM | 8.704E-05 | | .000 | | .000 | |  |
|  | Postcentral WM | -3.903E-05 | | .000 | | .174 | |  |
|  | Precentral WM | -9.190E-05 | | .000 | | .000 | |  |
|  | Cortical GM | -3.657E-06 | | .000 | | .000 | |  |
|  | Cortical WM | 1.648E-06 | | .000 | | .010 | |  |
|  | Age | .016 | | .009 | | .067 | |  |
|  | Sex | -.018 | | .031 | | .549 | |  |
|  | ICV | 5.878E-07 | | .000 | | .000 | |  |
| 60 | (Constant) | 1.939 | | .605 | | .001 | |  |
|  | Postcentral GM | -4.609E-05 | | .000 | | .092 | |  |
|  | Precentral GM | 6.432E-05 | | .000 | | .000 | |  |
|  | Postcentral WM | 5.204E-05 | | .000 | | .098 | |  |
|  | Precentral WM | -5.683E-05 | | .000 | | .000 | |  |
|  | Cortical GM | -2.402E-06 | | .000 | | .005 | |  |
|  | Cortical WM | -1.566E-06 | | .000 | | .017 | |  |
|  | Age | .003 | | .009 | | .769 | |  |
|  | Sex | -.114 | | .031 | | .000 | |  |
|  | ICV | 8.972E-07 | | .000 | | .000 | |  |
| 40+60 | (Constant) | 1.745 | | .115 | | .000 | |  |
|  | Postcentral GM | -2.088E-07 | | .000 | | .991 | |  |
|  | Precentral GM | 6.703E-05 | | .000 | | .000 | |  |
|  | Postcentral WM | -1.302E-06 | | .000 | | .950 | |  |
|  | Precentral WM | -7.092E-05 | | .000 | | .000 | |  |
|  | Cortical GM | -2.848E-06 | | .000 | | .000 | |  |
|  | Cortical WM | 1.050E-07 | | .000 | | .816 | |  |
|  | AgeG | .255 | | .023 | | .000 | |  |
|  | AgeC | .011 | | .006 | | .064 | |  |
|  | Sex | -.070 | | .022 | | .001 | |  |
|  | ICV | 7.085E-07 | | .000 | | .000 | |  |
| Right hemisphere | | | | | | | |  |
|  | | | | | | | | |
| Model | | | Unstandardized Coefficients | | | | Sig. | |
|  |  |  | B | | Std. Error | |  |  |
| 40 | (Constant) | | .827 | | .359 | | .022 | |
|  | Postcentral GM | | 3.255E-05 | | .000 | | .067 | |
|  | Precentral GM | | 1.009E-04 | | .000 | | .000 | |
|  | Postcentral WM | | -5.761E-05 | | .000 | | .007 | |
|  | Precentral WM | | -8.358E-05 | | .000 | | .000 | |
|  | Cortical GM | | -2.973E-06 | | .000 | | .000 | |
|  | Cortical WM | | 1.268E-06 | | .000 | | .011 | |
|  | Age | | .014 | | .007 | | .047 | |
|  | Sex | | -.027 | | .024 | | .269 | |
|  | ICV | | 5.153E-07 | | .000 | | .000 | |
| 60 | (Constant) | | 1.861 | | .589 | | .002 | |
|  | Postcentral GM | | 3.556E-05 | | .000 | | .142 | |
|  | Precentral GM | | 2.380E-05 | | .000 | | .157 | |
|  | Postcentral WM | | -4.994E-05 | | .000 | | .061 | |
|  | Precentral WM | | -4.847E-05 | | .000 | | .001 | |
|  | Cortical GM | | -2.018E-06 | | .000 | | .016 | |
|  | Cortical WM | | -2.833E-07 | | .000 | | .640 | |
|  | Age | | .000 | | .008 | | .963 | |
|  | Sex | | -.074 | | .030 | | .014 | |
|  | ICV | | 8.048E-07 | | .000 | | .000 | |
| 40+60 | (Constant) | | 1.615 | | .101 | | .000 | |
|  | Postcentral GM | | 3.328E-05 | | .000 | | .024 | |
|  | Precentral GM | | 6.120E-05 | | .000 | | .000 | |
|  | Postcentral WM | | -5.228E-05 | | .000 | | .002 | |
|  | Precentral WM | | -6.363E-05 | | .000 | | .000 | |
|  | Cortical GM | | -2.450E-06 | | .000 | | .000 | |
|  | Cortical WM | | 5.060E-07 | | .000 | | .195 | |
|  | AgeG | | .206 | | .020 | | .000 | |
|  | AgeC | | .008 | | .005 | | .121 | |
|  | Sex | | -.056 | | .019 | | .003 | |
|  | ICV | | 6.184E-07 | | .000 | | .000 | |

1. Lateral sulcus

Left hemisphere

| Model | | Unstandardized Coefficients | | Sig. |
| --- | --- | --- | --- | --- |
|  |  | B | Std. Error |  |
| 40 | (Constant) | .879 | .319 | .006 |
|  | Superior temporal GM | 3.200E-05 | .000 | .008 |
|  | Superior temporal WM | -6.303E-05 | .000 | .000 |
|  | Cortical GM | -2.357E-06 | .000 | .000 |
|  | Cortical WM | 3.542E-07 | .000 | .291 |
|  | Age | .006 | .006 | .341 |
|  | Sex | .019 | .021 | .371 |
|  | ICV | 5.552E-07 | .000 | .000 |
| 60 | (Constant) | 1.379 | .404 | .001 |
|  | Superior temporal GM | 9.030E-06 | .000 | .474 |
|  | Superior temporal WM | -2.107E-05 | .000 | .133 |
|  | Cortical GM | -1.659E-06 | .000 | .002 |
|  | Cortical WM | -2.403E-06 | .000 | .000 |
|  | Age | -.001 | .006 | .834 |
|  | Sex | -.029 | .021 | .159 |
|  | ICV | 1.154E-06 | .000 | .000 |
| 40+60 | (Constant) | 1.151 | .080 | .000 |
|  | Superior temporal GM | 2.196E-05 | .000 | .013 |
|  | Superior temporal WM | -4.410E-05 | .000 | .000 |
|  | Cortical GM | -1.864E-06 | .000 | .000 |
|  | Cortical WM | -9.442E-07 | .000 | .000 |
|  | AgeG | .162 | .014 | .000 |
|  | AgeC | .005 | .004 | .291 |
|  | Sex | -.008 | .015 | .615 |
|  | ICV | 8.008E-07 | .000 | .000 |

Right hemisphere

| Model | | Unstandardized Coefficients | | Sig. |
| --- | --- | --- | --- | --- |
|  |  | B | Std. Error |  |
| 40 | (Constant) | 1.178 | .295 | .000 |
|  | Superior temporal GM | 1.399E-05 | .000 | .292 |
|  | Superior temporal WM | -2.817E-05 | .000 | .128 |
|  | Cortical GM | -1.795E-06 | .000 | .000 |
|  | Cortical WM | -8.712E-08 | .000 | .798 |
|  | Age | .002 | .006 | .744 |
|  | Sex | .000 | .020 | .990 |
|  | ICV | 4.349E-07 | .000 | .000 |
| 60 | (Constant) | 1.312 | .389 | .001 |
|  | Superior temporal GM | 9.283E-07 | .000 | .944 |
|  | Superior temporal WM | -1.053E-05 | .000 | .529 |
|  | Cortical GM | -1.889E-06 | .000 | .000 |
|  | Cortical WM | -2.777E-06 | .000 | .000 |
|  | Age | .001 | .006 | .801 |
|  | Sex | -.017 | .020 | .388 |
|  | ICV | 1.270E-06 | .000 | .000 |
| 40+60 | (Constant) | 1.249 | .076 | .000 |
|  | Superior temporal GM | 8.107E-06 | .000 | .394 |
|  | Superior temporal WM | -1.893E-05 | .000 | .137 |
|  | Cortical GM | -1.510E-06 | .000 | .000 |
|  | Cortical WM | -1.366E-06 | .000 | .000 |
|  | AgeG | .144 | .014 | .000 |
|  | AgeC | .005 | .004 | .221 |
|  | Sex | -.009 | .014 | .538 |
|  | ICV | 7.602E-07 | .000 | .000 |

1. Superior temporal sulcus

Left hemisphere

| Model | | Unstandardized Coefficients | | Sig. |
| --- | --- | --- | --- | --- |
|  |  | B | Std. Error |  |
| 40 | (Constant) | 1.479 | .425 | .001 |
|  | Superior temporal GM | -1.380E-05 | .000 | .400 |
|  | Middle temporal GM | -4.109E-05 | .000 | .003 |
|  | Superior temporal WM | -9.886E-06 | .000 | .618 |
|  | Middle temporal WM | -2.716E-08 | .000 | .999 |
|  | Cortical GM | -2.260E-06 | .000 | .001 |
|  | Cortical WM | 6.770E-07 | .000 | .189 |
|  | Age | .000 | .008 | .974 |
|  | Sex | .037 | .028 | .191 |
|  | ICV | 6.358E-07 | .000 | .000 |
| 60 | (Constant) | 1.302 | .356 | .000 |
|  | Superior temporal GM | -1.823E-05 | .000 | .108 |
|  | Middle temporal GM | 5.168E-06 | .000 | .607 |
|  | Superior temporal WM | 1.894E-05 | .000 | .133 |
|  | Middle temporal WM | -3.533E-06 | .000 | .813 |
|  | Cortical GM | -1.621E-06 | .000 | .003 |
|  | Cortical WM | -1.863E-06 | .000 | .000 |
|  | Age | .002 | .005 | .752 |
|  | Sex | .001 | .018 | .942 |
|  | ICV | 9.245E-07 | .000 | .000 |
| 40+60 | (Constant) | 1.404 | .092 | .000 |
|  | Superior temporal GM | -1.586E-05 | .000 | .125 |
|  | Middle temporal GM | -2.188E-05 | .000 | .014 |
|  | Superior temporal WM | -1.054E-06 | .000 | .930 |
|  | Middle temporal WM | 1.440E-06 | .000 | .917 |
|  | Cortical GM | -1.824E-06 | .000 | .000 |
|  | Cortical WM | -5.853E-07 | .000 | .066 |
|  | AgeG | .091 | .016 | .000 |
|  | AgeC | .002 | .005 | .669 |
|  | Sex | .014 | .017 | .409 |
|  | ICV | 7.860E-07 | .000 | .000 |

Right hemisphere

| Model | | Unstandardized Coefficients | | Sig. |
| --- | --- | --- | --- | --- |
|  |  | B | Std. Error |  |
| 40 | (Constant) | 1.276 | .297 | .000 |
|  | Superior temporal GM | -3.297E-06 | .000 | .807 |
|  | Middle temporal GM | -4.159E-05 | .000 | .000 |
|  | Superior temporal WM | -2.193E-05 | .000 | .244 |
|  | Middle temporal WM | 2.883E-05 | .000 | .100 |
|  | Cortical GM | -7.022E-07 | .000 | .208 |
|  | Cortical WM | 5.939E-08 | .000 | .884 |
|  | Age | .002 | .006 | .760 |
|  | Sex | .019 | .020 | .340 |
|  | ICV | 3.547E-07 | .000 | .000 |
| 60 | (Constant) | 1.596 | .331 | .000 |
|  | Superior temporal GM | 4.302E-06 | .000 | .705 |
|  | Middle temporal GM | -1.260E-05 | .000 | .152 |
|  | Superior temporal WM | -1.761E-05 | .000 | .217 |
|  | Middle temporal WM | 2.011E-05 | .000 | .178 |
|  | Cortical GM | -1.760E-06 | .000 | .001 |
|  | Cortical WM | -1.708E-06 | .000 | .000 |
|  | Age | -.002 | .005 | .608 |
|  | Sex | -.019 | .017 | .262 |
|  | ICV | 9.391E-07 | .000 | .000 |
| 40+60 | (Constant) | 1.357 | .071 | .000 |
|  | Superior temporal GM | 9.797E-07 | .000 | .912 |
|  | Middle temporal GM | -2.967E-05 | .000 | .000 |
|  | Superior temporal WM | -2.107E-05 | .000 | .075 |
|  | Middle temporal WM | 2.868E-05 | .000 | .014 |
|  | Cortical GM | -9.834E-07 | .000 | .010 |
|  | Cortical WM | -8.003E-07 | .000 | .002 |
|  | AgeG | .087 | .013 | .000 |
|  | AgeC | .002 | .004 | .644 |
|  | Sex | -.002 | .013 | .863 |
|  | ICV | 5.863E-07 | .000 | .000 |

1. Intra-parietal sulcus

Left hemisphere

| Model | | Unstandardized Coefficients | | Sig. |
| --- | --- | --- | --- | --- |
|  |  | B | Std. Error |  |
| 40 | (Constant) | 1.073 | .482 | .027 |
|  | Superior parietal GM | 9.658E-06 | .000 | .600 |
|  | Inferior parietal GM | -3.751E-05 | .000 | .061 |
|  | Superior parietal WM | -1.493E-05 | .000 | .448 |
|  | Inferior parietal WM | 3.659E-05 | .000 | .070 |
|  | Cortical GM | -1.127E-06 | .000 | .195 |
|  | Cortical WM | -3.251E-07 | .000 | .650 |
|  | Age | .007 | .009 | .429 |
|  | Sex | -.024 | .033 | .470 |
|  | ICV | 3.838E-07 | .000 | .014 |
| 60 | (Constant) | .788 | .509 | .122 |
|  | Superior parietal GM | -4.707E-05 | .000 | .006 |
|  | Inferior parietal GM | -2.964E-06 | .000 | .852 |
|  | Superior parietal WM | 3.343E-05 | .000 | .034 |
|  | Inferior parietal WM | 1.617E-05 | .000 | .324 |
|  | Cortical GM | 7.848E-07 | .000 | .349 |
|  | Cortical WM | -2.867E-06 | .000 | .000 |
|  | Age | .011 | .007 | .124 |
|  | Sex | -.093 | .026 | .000 |
|  | ICV | 5.867E-07 | .000 | .000 |
| 40+60 | (Constant) | 1.436 | .112 | .000 |
|  | Superior parietal GM | -1.428E-05 | .000 | .260 |
|  | Inferior parietal GM | -2.056E-05 | .000 | .110 |
|  | Superior parietal WM | 5.569E-06 | .000 | .660 |
|  | Inferior parietal WM | 2.637E-05 | .000 | .045 |
|  | Cortical GM | -2.642E-07 | .000 | .662 |
|  | Cortical WM | -1.538E-06 | .000 | .001 |
|  | AgeG | .140 | .021 | .000 |
|  | AgeC | .010 | .006 | .084 |
|  | Sex | -.064 | .021 | .002 |
|  | ICV | 4.794E-07 | .000 | .000 |

Right hemisphere

| Model | | Unstandardized Coefficients | | Sig. |
| --- | --- | --- | --- | --- |
|  |  | B | Std. Error |  |
| 40 | (Constant) | 1.546 | .413 | .000 |
|  | Superior parietal GM | -9.135E-06 | .000 | .555 |
|  | Inferior parietal GM | -3.316E-05 | .000 | .022 |
|  | Superior parietal WM | 1.183E-05 | .000 | .473 |
|  | Inferior parietal WM | 3.188E-05 | .000 | .043 |
|  | Cortical GM | -1.171E-06 | .000 | .121 |
|  | Cortical WM | -8.967E-07 | .000 | .143 |
|  | Age | .002 | .008 | .842 |
|  | Sex | -.077 | .027 | .005 |
|  | ICV | 4.124E-07 | .000 | .002 |
| 60 | (Constant) | 1.512 | .468 | .001 |
|  | Superior parietal GM | -2.783E-05 | .000 | .039 |
|  | Inferior parietal GM | -1.865E-05 | .000 | .128 |
|  | Superior parietal WM | -7.304E-06 | .000 | .600 |
|  | Inferior parietal WM | 2.110E-05 | .000 | .103 |
|  | Cortical GM | 5.046E-07 | .000 | .516 |
|  | Cortical WM | -1.652E-06 | .000 | .002 |
|  | Age | .001 | .007 | .881 |
|  | Sex | -.087 | .024 | .000 |
|  | ICV | 4.895E-07 | .000 | .001 |
| 40+60 | (Constant) | 1.596 | .097 | .000 |
|  | Superior parietal GM | -1.626E-05 | .000 | .114 |
|  | Inferior parietal GM | -2.327E-05 | .000 | .014 |
|  | Superior parietal WM | 8.094E-07 | .000 | .940 |
|  | Inferior parietal WM | 2.402E-05 | .000 | .018 |
|  | Cortical GM | -5.308E-07 | .000 | .322 |
|  | Cortical WM | -1.234E-06 | .000 | .002 |
|  | AgeG | .056 | .018 | .002 |
|  | AgeC | .002 | .005 | .639 |
|  | Sex | -.085 | .018 | .000 |
|  | ICV | 4.693E-07 | .000 | .000 |

**Sulcal depth**

1. Superior frontal sulcus

Left hemisphere

| Model | | Unstandardized Coefficients | | Sig. |
| --- | --- | --- | --- | --- |
|  |  | B | Std. Error |  |
| 40 | (Constant) | 11.599 | 2.681 | .000 |
|  | Superior frontal GM | .000 | .000 | .059 |
|  | Middle frontal GM | -2.013E-05 | .000 | .838 |
|  | Superior frontal WM | 8.365E-05 | .000 | .355 |
|  | Middle frontal WM | 8.238E-05 | .000 | .428 |
|  | Cortical GM | 8.515E-06 | .000 | .107 |
|  | Cortical WM | -8.690E-06 | .000 | .044 |
|  | Age | .040 | .052 | .439 |
|  | Sex | .034 | .179 | .848 |
|  | ICV | -3.607E-06 | .000 | .000 |
| 60 | (Constant) | 6.679 | 3.653 | .068 |
|  | Superior frontal GM | .000 | .000 | .150 |
|  | Middle frontal GM | -4.293E-06 | .000 | .964 |
|  | Superior frontal WM | 3.648E-05 | .000 | .706 |
|  | Middle frontal WM | 4.253E-05 | .000 | .679 |
|  | Cortical GM | 1.228E-05 | .000 | .074 |
|  | Cortical WM | 4.049E-06 | .000 | .444 |
|  | Age | .096 | .052 | .067 |
|  | Sex | -.015 | .184 | .935 |
|  | ICV | -6.812E-06 | .000 | .000 |
| 40+60 | (Constant) | 13.284 | .697 | .000 |
|  | Superior frontal GM | .000 | .000 | .037 |
|  | Middle frontal GM | -5.945E-06 | .000 | .929 |
|  | Superior frontal WM | 8.007E-05 | .000 | .221 |
|  | Middle frontal WM | 6.212E-05 | .000 | .389 |
|  | Cortical GM | 9.308E-06 | .000 | .025 |
|  | Cortical WM | -3.501E-06 | .000 | .293 |
|  | AgeG | .202 | .127 | .112 |
|  | AgeC | .055 | .036 | .130 |
|  | Sex | .009 | .128 | .942 |
|  | ICV | -4.766E-06 | .000 | .000 |

Right hemisphere

| Model | | Unstandardized Coefficients | | Sig. |
| --- | --- | --- | --- | --- |
|  |  | B | Std. Error |  |
| 40 | (Constant) | 10.963 | 3.006 | .000 |
|  | Superior frontal GM | .000 | .000 | .085 |
|  | Middle frontal GM | .000 | .000 | .129 |
|  | Superior frontal WM | .000 | .000 | .307 |
|  | Middle frontal WM | -3.794E-05 | .000 | .701 |
|  | Cortical GM | 1.125E-05 | .000 | .067 |
|  | Cortical WM | -4.196E-06 | .000 | .353 |
|  | Age | .068 | .058 | .243 |
|  | Sex | -.062 | .198 | .756 |
|  | ICV | -3.249E-06 | .000 | .001 |
| 60 | (Constant) | 8.011 | 3.672 | .030 |
|  | Superior frontal GM | 4.455E-05 | .000 | .610 |
|  | Middle frontal GM | -1.638E-05 | .000 | .864 |
|  | Superior frontal WM | .000 | .000 | .232 |
|  | Middle frontal WM | -9.784E-05 | .000 | .350 |
|  | Cortical GM | 1.870E-05 | .000 | .009 |
|  | Cortical WM | 8.813E-06 | .000 | .063 |
|  | Age | .072 | .052 | .172 |
|  | Sex | .161 | .184 | .382 |
|  | ICV | -7.704E-06 | .000 | .000 |
| 40+60 | (Constant) | 13.493 | .744 | .000 |
|  | Superior frontal GM | 9.658E-05 | .000 | .132 |
|  | Middle frontal GM | -6.139E-05 | .000 | .362 |
|  | Superior frontal WM | .000 | .000 | .100 |
|  | Middle frontal WM | -8.870E-05 | .000 | .214 |
|  | Cortical GM | 1.220E-05 | .000 | .008 |
|  | Cortical WM | 2.209E-06 | .000 | .499 |
|  | AgeG | .283 | .132 | .033 |
|  | AgeC | .058 | .039 | .139 |
|  | Sex | .047 | .136 | .732 |
|  | ICV | -4.869E-06 | .000 | .000 |

1. Central sulcus

Left hemisphere

| Model | | Unstandardized Coefficients | | Sig. |
| --- | --- | --- | --- | --- |
|  |  | B | Std. Error |  |
| 40 | (Constant) | 1.49E+01 | 2.439 | .000 |
|  | Postcentral GM | -2.47E-04 | .000 | .050 |
|  | Precentral GM | 1.16E-04 | .000 | .246 |
|  | Postcentral WM | 2.31E-04 | .000 | .128 |
|  | Precentral WM | 1.89E-04 | .000 | .032 |
|  | Cortical GM | 1.36E-05 | .000 | .000 |
|  | Cortical WM | -5.17E-06 | .000 | .126 |
|  | Age | 4.89E-02 | .047 | .300 |
|  | Sex | 1.44E-02 | .162 | .929 |
|  | ICV | -5.80E-06 | .000 | .000 |
| 60 | (Constant) | 2.03E+01 | 3.174 | .000 |
|  | Postcentral GM | 1.78E-04 | .000 | .214 |
|  | Precentral GM | 1.77E-04 | .000 | .062 |
|  | Postcentral WM | -1.65E-04 | .000 | .317 |
|  | Precentral WM | 2.47E-04 | .000 | .002 |
|  | Cortical GM | 9.22E-07 | .000 | .837 |
|  | Cortical WM | -2.69E-06 | .000 | .430 |
|  | Age | -2.63E-02 | .045 | .564 |
|  | Sex | -2.47E-01 | .162 | .129 |
|  | ICV | -5.21E-06 | .000 | .000 |
| 40+60 | (Constant) | 1.76E+01 | .604 | .000 |
|  | Postcentral GM | -6.24E-05 | .000 | .501 |
|  | Precentral GM | 1.49E-04 | .000 | .028 |
|  | Postcentral WM | 3.84E-05 | .000 | .726 |
|  | Precentral WM | 2.22E-04 | .000 | .000 |
|  | Cortical GM | 8.69E-06 | .000 | .002 |
|  | Cortical WM | -3.85E-06 | .000 | .103 |
|  | AgeG | 1.42E-01 | .119 | .234 |
|  | AgeC | 9.80E-03 | .032 | .763 |
|  | Sex | -7.92E-02 | .114 | .487 |
|  | ICV | -5.71E-06 | .000 | .000 |

Right hemisphere

| Model | | Unstandardized Coefficients | | Sig. |
| --- | --- | --- | --- | --- |
|  |  | B | Std. Error |  |
| 40 | (Constant) | 1.28E+01 | 2.386 | .000 |
|  | Postcentral GM | 3.37E-05 | .000 | .775 |
|  | Precentral GM | -1.38E-04 | .000 | .137 |
|  | Postcentral WM | -7.06E-05 | .000 | .620 |
|  | Precentral WM | 3.47E-04 | .000 | .000 |
|  | Cortical GM | 1.60E-05 | .000 | .000 |
|  | Cortical WM | -6.71E-06 | .000 | .043 |
|  | Age | 1.10E-01 | .046 | .018 |
|  | Sex | -2.00E-01 | .159 | .210 |
|  | ICV | -5.96E-06 | .000 | .000 |
| 60 | (Constant) | 1.94E+01 | 3.002 | .000 |
|  | Postcentral GM | 2.19E-04 | .000 | .077 |
|  | Precentral GM | 2.19E-04 | .000 | .011 |
|  | Postcentral WM | -2.14E-04 | .000 | .114 |
|  | Precentral WM | 6.04E-05 | .000 | .396 |
|  | Cortical GM | -5.37E-06 | .000 | .209 |
|  | Cortical WM | 3.14E-06 | .000 | .310 |
|  | Age | 9.60E-03 | .043 | .823 |
|  | Sex | -4.14E-01 | .153 | .007 |
|  | ICV | -4.64E-06 | .000 | .000 |
| 40+60 | (Constant) | 1.86E+01 | .586 | .000 |
|  | Postcentral GM | 8.81E-05 | .000 | .300 |
|  | Precentral GM | 3.18E-05 | .000 | .612 |
|  | Postcentral WM | -1.11E-04 | .000 | .260 |
|  | Precentral WM | 2.10E-04 | .000 | .000 |
|  | Cortical GM | 8.42E-06 | .000 | .001 |
|  | Cortical WM | -1.96E-06 | .000 | .387 |
|  | AgeG | 3.63E-02 | .115 | .752 |
|  | AgeC | 5.79E-02 | .032 | .067 |
|  | Sex | -2.54E-01 | .111 | .022 |
|  | ICV | -5.84E-06 | .000 | .000 |

1. Lateral sulcus

Left hemisphere

| Model | | Unstandardized Coefficients | | Sig. |
| --- | --- | --- | --- | --- |
|  |  | B | Std. Error |  |
| 40 | (Constant) | 1.25E+01 | 5.388 | .020 |
|  | Superior temporal GM | -2.73E-04 | .000 | .181 |
|  | Superior temporal WM | 7.57E-04 | .000 | .003 |
|  | Cortical GM | 2.11E-05 | .000 | .006 |
|  | Cortical WM | -8.94E-06 | .000 | .115 |
|  | Age | 1.77E-01 | .103 | .088 |
|  | Sex | -1.78E-01 | .353 | .615 |
|  | ICV | -5.54E-06 | .000 | .001 |
| 60 | (Constant) | 1.69E+01 | 7.232 | .020 |
|  | Superior temporal GM | -4.30E-04 | .000 | .057 |
|  | Superior temporal WM | 1.23E-03 | .000 | .000 |
|  | Cortical GM | 2.56E-05 | .000 | .007 |
|  | Cortical WM | -3.20E-06 | .000 | .599 |
|  | Age | 5.36E-02 | .103 | .604 |
|  | Sex | 2.67E-01 | .370 | .471 |
|  | ICV | -9.51E-06 | .000 | .000 |
| 40+60 | (Constant) | 2.09E+01 | 1.352 | .000 |
|  | Superior temporal GM | -3.63E-04 | .000 | .016 |
|  | Superior temporal WM | 1.01E-03 | .000 | .000 |
|  | Cortical GM | 2.12E-05 | .000 | .000 |
|  | Cortical WM | -6.07E-06 | .000 | .142 |
|  | AgeG | 7.28E-02 | .246 | .767 |
|  | AgeC | 9.52E-02 | .072 | .189 |
|  | Sex | 1.94E-02 | .254 | .939 |
|  | ICV | -7.17E-06 | .000 | .000 |

Right hemisphere

| Model | | Unstandardized Coefficients | | Sig. |
| --- | --- | --- | --- | --- |
|  |  | B | Std. Error |  |
| 40 | (Constant) | 2.03E+01 | 4.844 | .000 |
|  | Superior temporal GM | -4.58E-04 | .000 | .036 |
|  | Superior temporal WM | 1.21E-03 | .000 | .000 |
|  | Cortical GM | 1.73E-05 | .000 | .025 |
|  | Cortical WM | -1.28E-05 | .000 | .022 |
|  | Age | -3.72E-02 | .093 | .690 |
|  | Sex | 5.87E-01 | .321 | .069 |
|  | ICV | -2.87E-06 | .000 | .069 |
| 60 | (Constant) | 1.37E+01 | 6.700 | .042 |
|  | Superior temporal GM | -4.60E-04 | .000 | .044 |
|  | Superior temporal WM | 1.15E-03 | .000 | .000 |
|  | Cortical GM | 1.08E-05 | .000 | .229 |
|  | Cortical WM | -1.04E-05 | .000 | .074 |
|  | Age | 1.05E-01 | .095 | .274 |
|  | Sex | 3.06E-01 | .337 | .364 |
|  | ICV | -3.15E-06 | .000 | .129 |
| 40+60 | (Constant) | 1.97E+01 | 1.244 | .000 |
|  | Superior temporal GM | -4.65E-04 | .000 | .003 |
|  | Superior temporal WM | 1.19E-03 | .000 | .000 |
|  | Cortical GM | 1.51E-05 | .000 | .009 |
|  | Cortical WM | -1.18E-05 | .000 | .003 |
|  | AgeG | -4.57E-01 | .228 | .045 |
|  | AgeC | 3.38E-02 | .066 | .608 |
|  | Sex | 4.68E-01 | .231 | .044 |
|  | ICV | -3.13E-06 | .000 | .011 |

1. Superior temporal sulcus

Left hemisphere

| Model | | Unstandardized Coefficients | | Sig. |
| --- | --- | --- | --- | --- |
|  |  | B | Std. Error |  |
| 40 | (Constant) | 1.53E+01 | 2.873 | .000 |
|  | Superior temporal GM | 4.32E-04 | .000 | .000 |
|  | Middle temporal GM | 6.06E-04 | .000 | .000 |
|  | Superior temporal WM | -9.60E-04 | .000 | .000 |
|  | Middle temporal WM | -2.32E-04 | .000 | .128 |
|  | Cortical GM | 1.27E-06 | .000 | .782 |
|  | Cortical WM | 1.15E-05 | .000 | .001 |
|  | Age | -3.45E-02 | .055 | .532 |
|  | Sex | 3.62E-01 | .189 | .057 |
|  | ICV | -4.42E-06 | .000 | .000 |
| 60 | (Constant) | 1.71E+01 | 3.994 | .000 |
|  | Superior temporal GM | 2.63E-04 | .000 | .039 |
|  | Middle temporal GM | 6.63E-04 | .000 | .000 |
|  | Superior temporal WM | -8.18E-04 | .000 | .000 |
|  | Middle temporal WM | -4.60E-04 | .000 | .006 |
|  | Cortical GM | -3.00E-06 | .000 | .623 |
|  | Cortical WM | 1.72E-05 | .000 | .000 |
|  | Age | -1.86E-02 | .057 | .743 |
|  | Sex | 8.04E-02 | .204 | .693 |
|  | ICV | -4.95E-06 | .000 | .000 |
| 40+60 | (Constant) | 1.44E+01 | .741 | .000 |
|  | Superior temporal GM | 3.53E-04 | .000 | .000 |
|  | Middle temporal GM | 6.27E-04 | .000 | .000 |
|  | Superior temporal WM | -8.69E-04 | .000 | .000 |
|  | Middle temporal WM | -3.30E-04 | .000 | .003 |
|  | Cortical GM | -5.71E-07 | .000 | .875 |
|  | Cortical WM | 1.41E-05 | .000 | .000 |
|  | AgeG | 5.73E-01 | .133 | .000 |
|  | AgeC | -3.42E-02 | .039 | .383 |
|  | Sex | 2.47E-01 | .138 | .074 |
|  | ICV | -4.81E-06 | .000 | .000 |

Right hemisphere

| 40 | (Constant) | 1.35E+01 | 3.030 | .000 |
| --- | --- | --- | --- | --- |
|  | Superior temporal GM | 1.06E-04 | .000 | .442 |
|  | Middle temporal GM | 6.78E-04 | .000 | .000 |
|  | Superior temporal WM | -6.01E-04 | .000 | .002 |
|  | Middle temporal WM | -3.25E-04 | .000 | .069 |
|  | Cortical GM | -3.32E-07 | .000 | .954 |
|  | Cortical WM | 1.13E-05 | .000 | .007 |
|  | Age | 6.04E-02 | .058 | .301 |
|  | Sex | 4.46E-01 | .203 | .028 |
|  | ICV | -4.75E-06 | .000 | .000 |
| 60 | (Constant) | 1.17E+01 | 4.034 | .004 |
|  | Superior temporal GM | -4.50E-05 | .000 | .745 |
|  | Middle temporal GM | 3.96E-04 | .000 | .000 |
|  | Superior temporal WM | -5.19E-04 | .000 | .003 |
|  | Middle temporal WM | 3.46E-05 | .000 | .849 |
|  | Cortical GM | 1.05E-05 | .000 | .092 |
|  | Cortical WM | 9.08E-06 | .000 | .023 |
|  | Age | 9.18E-02 | .058 | .111 |
|  | Sex | 2.01E-01 | .203 | .322 |
|  | ICV | -6.51E-06 | .000 | .000 |
| 40+60 | (Constant) | 1.69E+01 | .771 | .000 |
|  | Superior temporal GM | 2.68E-05 | .000 | .780 |
|  | Middle temporal GM | 5.47E-04 | .000 | .000 |
|  | Superior temporal WM | -5.32E-04 | .000 | .000 |
|  | Middle temporal WM | -1.46E-04 | .000 | .245 |
|  | Cortical GM | 4.38E-06 | .000 | .287 |
|  | Cortical WM | 9.59E-06 | .000 | .001 |
|  | AgeG | 2.79E-01 | .142 | .049 |
|  | AgeC | 7.30E-02 | .040 | .072 |
|  | Sex | 3.30E-01 | .142 | .021 |
|  | ICV | -5.44E-06 | .000 | .000 |

1. Intra-parietal sulcus

Left hemisphere

| Model | | Unstandardized Coefficients | | Sig. |
| --- | --- | --- | --- | --- |
|  |  | B | Std. Error |  |
| 40 | (Constant) | 1.10E+01 | 2.501 | .000 |
|  | Superior parietal GM | -3.21E-04 | .000 | .001 |
|  | Inferior parietal GM | 2.31E-04 | .000 | .027 |
|  | Superior parietal WM | 4.74E-04 | .000 | .000 |
|  | Inferior parietal WM | -2.28E-04 | .000 | .030 |
|  | Cortical GM | 1.70E-05 | .000 | .000 |
|  | Cortical WM | -5.15E-06 | .000 | .166 |
|  | Age | 1.05E-01 | .048 | .031 |
|  | Sex | 2.57E-01 | .170 | .130 |
|  | ICV | -5.31E-06 | .000 | .000 |
| 60 | (Constant) | 1.93E+01 | 3.149 | .000 |
|  | Superior parietal GM | -1.81E-04 | .000 | .084 |
|  | Inferior parietal GM | 2.17E-04 | .000 | .028 |
|  | Superior parietal WM | 3.05E-04 | .000 | .002 |
|  | Inferior parietal WM | -1.94E-04 | .000 | .057 |
|  | Cortical GM | 6.75E-06 | .000 | .193 |
|  | Cortical WM | 1.93E-06 | .000 | .575 |
|  | Age | -3.93E-02 | .044 | .377 |
|  | Sex | 1.52E-01 | .160 | .341 |
|  | ICV | -4.88E-06 | .000 | .000 |
| 40+60 | (Constant) | 1.62E+01 | .617 | .000 |
|  | Superior parietal GM | -2.68E-04 | .000 | .000 |
|  | Inferior parietal GM | 2.20E-04 | .000 | .002 |
|  | Superior parietal WM | 4.08E-04 | .000 | .000 |
|  | Inferior parietal WM | -2.06E-04 | .000 | .005 |
|  | Cortical GM | 1.30E-05 | .000 | .000 |
|  | Cortical WM | -1.74E-06 | .000 | .492 |
|  | AgeG | 9.40E-02 | .114 | .409 |
|  | AgeC | 3.22E-02 | .033 | .324 |
|  | Sex | 2.22E-01 | .116 | .056 |
|  | ICV | -5.31E-06 | .000 | .000 |

Right hemisphere

| Model | | Unstandardized Coefficients | | Sig. |
| --- | --- | --- | --- | --- |
|  |  | B | Std. Error |  |
| 40 | (Constant) | 1.32E+01 | 2.515 | .000 |
|  | Superior parietal GM | -3.10E-04 | .000 | .001 |
|  | Inferior parietal GM | 1.80E-04 | .000 | .041 |
|  | Superior parietal WM | 5.61E-04 | .000 | .000 |
|  | Inferior parietal WM | -2.10E-04 | .000 | .028 |
|  | Cortical GM | 1.74E-05 | .000 | .000 |
|  | Cortical WM | -8.79E-06 | .000 | .019 |
|  | Age | 4.58E-02 | .049 | .346 |
|  | Sex | 2.84E-01 | .167 | .089 |
|  | ICV | -4.65E-06 | .000 | .000 |
| 60 | (Constant) | 1.67E+01 | 2.916 | .000 |
|  | Superior parietal GM | -3.52E-04 | .000 | .000 |
|  | Inferior parietal GM | 2.12E-04 | .000 | .006 |
|  | Superior parietal WM | 5.42E-04 | .000 | .000 |
|  | Inferior parietal WM | -2.30E-04 | .000 | .004 |
|  | Cortical GM | 1.24E-05 | .000 | .010 |
|  | Cortical WM | -5.33E-06 | .000 | .101 |
|  | Age | -1.30E-02 | .041 | .754 |
|  | Sex | 2.80E-01 | .148 | .059 |
|  | ICV | -4.31E-06 | .000 | .000 |
| 40+60 | (Constant) | 1.56E+01 | .591 | .000 |
|  | Superior parietal GM | -3.37E-04 | .000 | .000 |
|  | Inferior parietal GM | 1.92E-04 | .000 | .001 |
|  | Superior parietal WM | 5.57E-04 | .000 | .000 |
|  | Inferior parietal WM | -2.16E-04 | .000 | .001 |
|  | Cortical GM | 1.56E-05 | .000 | .000 |
|  | Cortical WM | -7.02E-06 | .000 | .004 |
|  | AgeG | -3.47E-02 | .108 | .749 |
|  | AgeC | 1.48E-02 | .032 | .640 |
|  | Sex | 2.84E-01 | .111 | .010 |
|  | ICV | -4.66E-06 | .000 | .000 |

**Table 5. Contribution of local and global grey and white matter volumes to sulcal measures.** Sulci: **s**uperior frontal sulcus (frontal); central sulcus (central), lateral sulcus (lateral); superior temporal sulcus (temporal); intra-parietal sulcus (parietal). The significance level of R-squared changes was set at p < 0.01 as previously mentioned. The significant results are shown in green. The higher R square changes are shown in yellow.

Sulcal width

Left hemisphere

|  | Age group | Model 0 | Model 1 LGM | | Model 2 LWM | | Model 3 GGM | | Model 4 GWM | |
| --- | --- | --- | --- | --- | --- | --- | --- | --- | --- | --- |
| Sulcal width | | Adjusted R Square | R Square Change | Sig | R Square Change | Sig | R Square Change | Sig | R Square Change | Sig |
| Frontal | 40 | -.006 | .068 | .000 | .003 | .244 | .003 | .241 | .008 | .067 |
|  | 60 | .012 | .030 | .001 | .047 | .000 | .052 | .000 | .005 | .122 |
|  | 40+60 | .185 | .041 | .000 | .016 | .000 | .012 | .000 | .000 | .692 |
| Central | 40 | .006 | .006 | .117 | .108 | .000 | .046 | .000 | .012 | .019 |
|  | 60 | .010 | .001 | .554 | .062 | .000 | .029 | .000 | .011 | .029 |
|  | 40+60 | .255 | .003 | .078 | .062 | .000 | .027 | .000 | .000 | .968 |
| Lateral | 40 | .015 | .039 | .000 | .032 | .000 | .060 | .000 | .001 | .581 |
|  | 60 | .014 | .029 | .001 | .092 | .000 | .067 | .000 | .126 | .000 |
|  | 40+60 | .353 | .022 | .000 | .040 | .000 | .039 | .000 | .017 | .000 |
| Temporal | 40 | .015 | .144 | .000 | .001 | .454 | .017 | .004 | .003 | .259 |
|  | 60 | .014 | .047 | .000 | .038 | .000 | .092 | .000 | .073 | .000 |
|  | 40+60 | .209 | .082 | .000 | .003 | .055 | .029 | .000 | .005 | .014 |
| Parietal | 40 | .008 | .037 | .000 | .004 | .207 | .017 | .008 | .002 | .420 |
|  | 60 | .051 | .024 | .002 | .012 | .027 | .005 | .156 | .055 | .000 |
|  | 40+60 | .173 | .027 | .000 | .000 | .614 | .010 | .002 | .013 | .000 |

Right hemisphere

|  | Age group | Model 0 | Model 1 LGM | | Model 2 LWM | | Model 3 GGM | | Model 4 GWM | |
| --- | --- | --- | --- | --- | --- | --- | --- | --- | --- | --- |
| Sulcal width | | Adjusted R Square | R Square Change | Sig | R Square Change | Sig | R Square Change | Sig | R Square Change | Sig |
| Frontal | 40 | -.006 | .052 | .000 | .005 | .158 | .010 | .041 | .008 | .062 |
|  | 60 | .012 | .032 | .000 | .031 | .000 | .055 | .000 | .013 | .018 |
|  | 40+60 | .185 | .035 | .000 | .012 | .000 | .018 | .000 | .000 | .870 |
| Central | 40 | .006 | .000 | .745 | .123 | .000 | .053 | .000 | .006 | .076 |
|  | 60 | .010 | .001 | .449 | .093 | .000 | .032 | .000 | .002 | .307 |
|  | 40+60 | .255 | .001 | .387 | .079 | .000 | .029 | .000 | .000 | .607 |
| Lateral | 40 | .015 | .039 | .000 | .006 | .114 | .065 | .000 | .000 | .875 |
|  | 60 | .014 | .006 | .130 | .059 | .000 | .095 | .000 | .147 | .000 |
|  | 40+60 | .353 | .013 | .000 | .018 | .000 | .051 | .000 | .024 | .000 |
| Temporal | 40 | .015 | .131 | .000 | .002 | .308 | .015 | .008 | .001 | .585 |
|  | 60 | .014 | .039 | .000 | .038 | .000 | .100 | .000 | .073 | .000 |
|  | 40+60 | .209 | .074 | .000 | .003 | .088 | .031 | .000 | .007 | .006 |
| Parietal | 40 | .008 | .041 | .000 | .008 | .061 | .011 | .028 | .004 | .170 |
|  | 60 | .051 | .043 | .000 | .016 | .008 | .000 | .644 | .050 | .000 |
|  | 40+60 | .173 | .037 | .000 | .000 | .783 | .005 | .027 | .016 | .000 |

Sulcal depth

Left hemisphere

|  | Age group | Model 0 | Model 1 LGM | | Model 2 LWM | | Model 3 GGM | | Model 4 GWM | |
| --- | --- | --- | --- | --- | --- | --- | --- | --- | --- | --- |
| Sulcal depth | | Adjusted R Square | R Square Change | Sig | R Square Change | Sig | R Square Change | Sig | R Square Change | Sig |
| Frontal | 40 | -.006 | .070 | .000 | .001 | .520 | .011 | .027 | .009 | .051 |
|  | 60 | .012 | .096 | .000 | .019 | .004 | .028 | .000 | .004 | .159 |
|  | 40+60 | .185 | .079 | .000 | .002 | .178 | .015 | .000 | .000 | .525 |
| Central | 40 | .006 | .061 | .000 | .010 | .032 | .045 | .000 | .003 | .209 |
|  | 60 | .010 | .041 | .000 | .002 | .398 | .000 | .917 | .000 | .885 |
|  | 40+60 | .255 | .050 | .000 | .005 | .030 | .016 | .000 | .000 | .535 |
| Lateral | 40 | .015 | .012 | .028 | .018 | .006 | .022 | .003 | .007 | .097 |
|  | 60 | .014 | .010 | .052 | .073 | .000 | .011 | .032 | .003 | .282 |
|  | 40+60 | .353 | .010 | .004 | .043 | .000 | .014 | .000 | .005 | .043 |
| Temporal | 40 | .015 | .120 | .000 | .028 | .000 | .008 | .043 | .018 | .002 |
|  | 60 | .014 | .064 | .000 | .018 | .005 | .006 | .103 | .034 | .000 |
|  | 40+60 | .209 | .092 | .000 | .022 | .000 | .007 | .008 | .024 | .000 |
| Parietal | 40 | .008 | .046 | .000 | .000 | .658 | .036 | .000 | .228 | .634 |
|  | 60 | .051 | .022 | .002 | .005 | .132 | .012 | .019 | .000 | .700 |
|  | 40+60 | .173 | .035 | .000 | .001 | .407 | .024 | .000 | .000 | .910 |

Right hemisphere

|  | Age group | Model 0 | Model 1 LGM | | Model 2 LWM | | Model 3 GGM | | Model 4 GWM | |
| --- | --- | --- | --- | --- | --- | --- | --- | --- | --- | --- |
| Sulcal depth | | Adjusted R Square | R Square Change | Sig | R Square Change | Sig | R Square Change | Sig | R Square Change | Sig |
| Frontal | 40 | -.006 | .078 | .000 | .002 | .303 | .007 | .081 | .003 | .254 |
|  | 60 | .012 | .087 | .000 | .006 | .105 | .036 | .000 | .014 | .012 |
|  | 40+60 | .185 | .080 | .000 | .000 | .739 | .014 | .001 | .001 | .430 |
| Central | 40 | .006 | .049 | .000 | .022 | .001 | .047 | .000 | .008 | .046 |
|  | 60 | .010 | .050 | .000 | .000 | .915 | .001 | .469 | .004 | .198 |
|  | 40+60 | .255 | .049 | .000 | .006 | .020 | .015 | .000 | .001 | .452 |
| Lateral | 40 | .015 | .022 | .003 | .021 | .003 | .017 | .007 | .014 | .014 |
|  | 60 | .014 | .002 | .384 | .049 | .000 | .017 | .008 | .001 | .552 |
|  | 40+60 | .353 | .011 | .004 | .034 | .000 | .016 | .000 | .006 | .028 |
| Temporal | 40 | .015 | .126 | .000 | .003 | .199 | .004 | .189 | .005 | .134 |
|  | 60 | .014 | .047 | .000 | .002 | .311 | .016 | .008 | .009 | .043 |
|  | 40+60 | .209 | .087 | .000 | .003 | .116 | .008 | .005 | .006 | .019 |
| Parietal | 40 | .008 | .050 | .000 | .001 | .462 | .040 | .000 | .013 | .012 |
|  | 60 | .051 | .015 | .009 | .012 | .020 | .021 | .002 | .001 | .494 |
|  | 40+60 | .173 | .032 | .000 | .005 | .022 | .034 | .000 | .006 | .018 |

**Table 6. Contribution of local and global factors to sulcal measures.** Sulci: **s**uperior frontal sulcus (frontal); central sulcus (central), lateral sulcus (lateral); superior temporal sulcus (temporal); intra-parietal sulcus (parietal). The significance level of R-squared changes was set at p < 0.01 as previously mentioned. The significant results are shown in green. The higher R square changes are shown in yellow.

Sulcal width

|  | Age group | Model 0 | Model 1 LF (left) | | Model 2 GF (left) | | Model 1 LF (right) | | Model 2 GF (right) | |
| --- | --- | --- | --- | --- | --- | --- | --- | --- | --- | --- |
| Sulcal width | | Adjusted R Square | R Square Change | Sig | R Square Change | Sig | R Square Change | Sig | R Square Change | Sig |
| Frontal | 40 | -.006 | .060 | .000 | .008 | .178 | .070 | .000 | .012 | .067 |
|  | 60 | .012 | .026 | .020 | .025 | .003 | .058 | .000 | .022 | .007 |
|  | 40+60 | .185 | .031 | .000 | .002 | .410 | .055 | .000 | .003 | .187 |
| Central | 40 | .006 | .091 | .000 | .055 | .000 | .166 | .000 | .063 | .000 |
|  | 60 | .010 | .040 | .002 | .050 | .000 | .042 | .001 | .018 | .019 |
|  | 40+60 | .255 | .040 | .000 | .028 | .000 | .066 | .000 | .039 | .000 |
| Lateral | 40 | .015 | .039 | .000 | .062 | .000 | .005 | .313 | .049 | .000 |
|  | 60 | .014 | .005 | .316 | .150 | .000 | .001 | .764 | .207 | .000 |
|  | 40+60 | .353 | .013 | .000 | .049 | .000 | .002 | .325 | .065 | .000 |
| Temporal | 40 | .015 | .032 | .005 | .023 | .004 | .049 | .000 | .004 | .384 |
|  | 60 | .014 | .008 | .461 | .118 | .000 | .008 | .387 | .122 | .000 |
|  | 40+60 | .209 | .011 | .019 | .028 | .000 | .023 | .000 | .026 | .000 |
| Parietal | 40 | .008 | .011 | .353 | .009 | .152 | .013 | .225 | .025 | .005 |
|  | 60 | .051 | .025 | .027 | .064 | .000 | .035 | .005 | .025 | .004 |
|  | 40+60 | .173 | .006 | .178 | .018 | .000 | .011 | .027 | .020 | .000 |

Sulcal depth

|  | Age group | Model 0 | Model 1 LF (left) | | Model 2 GF (left) | | Model 1 LF (right) | | Model 2 GF (right) | |
| --- | --- | --- | --- | --- | --- | --- | --- | --- | --- | --- |
| Sulcal depth | | Adjusted R Square | R Square Change | Sig | R Square Change | Sig | R Square Change | Sig | R Square Change | Sig |
| Frontal | 40 | -.006 | .041 | .001 | .010 | .098 | .060 | .000 | .008 | .186 |
|  | 60 | .012 | .016 | .138 | .015 | .044 | .022 | .051 | .044 | .000 |
|  | 40+60 | .185 | .027 | .000 | .006 | .080 | .038 | .000 | .013 | .003 |
| Central | 40 | .006 | .057 | .000 | .019 | .019 | .045 | .000 | .016 | .034 |
|  | 60 | .010 | .075 | .000 | .017 | .026 | .049 | .000 | .009 | .163 |
|  | 40+60 | .255 | .057 | .000 | .016 | .001 | .033 | .000 | .013 | .004 |
| Lateral | 40 | .015 | .023 | .009 | .019 | .019 | .041 | .000 | .016 | .034 |
|  | 60 | .014 | .060 | .000 | .017 | .026 | .040 | .000 | .009 | .163 |
|  | 40+60 | .353 | .041 | .000 | .016 | .001 | .041 | .000 | .013 | .004 |
| Temporal | 40 | .015 | .186 | .000 | .026 | .001 | .120 | .000 | .019 | .008 |
|  | 60 | .014 | .166 | .000 | .042 | .000 | .085 | .000 | .026 | .002 |
|  | 40+60 | .209 | .170 | .000 | .033 | .000 | .099 | .000 | .020 | .000 |
| Parietal | 40 | .008 | .056 | .000 | .031 | .001 | .083 | .000 | .029 | .001 |
|  | 60 | .051 | .033 | .005 | .008 | .184 | .101 | .000 | .014 | .033 |
|  | 40+60 | .173 | .046 | .000 | .018 | .000 | .089 | .000 | .022 | .000 |

# Table 7. Sensitivity of sulcal width and depth to local brain volumes. Sulci: superior frontal sulcus (frontal); central sulcus (central), lateral sulcus (lateral); superior temporal sulcus (temporal); intra-parietal sulcus (parietal). The significance level of R-squared changes was set at p < 0.01 as previously mentioned. The significant results are shown in green. The higher R square changes are shown in yellow.

|  | Age group | Model 0 | Width (left) | | Depth (left) | | Model 0 | Width (right) | | Depth (right) | |
| --- | --- | --- | --- | --- | --- | --- | --- | --- | --- | --- | --- |
|  | | Adjusted R Square | R Square Change | Sig | R Square Change | Sig | Adjusted R Square | R Square Change | Sig | R Square Change | Sig |
| Frontal | 40 | .701 | .010 | .000 | .012 | .000 | .701 | .025 | .000 | .007 | .001 |
|  | 60 | .766 | .015 | .000 | .011 | .000 | .771 | .020 | .000 | .010 | .000 |
|  | 40+60 | .744 | .012 | .000 | .011 | .000 | .745 | .021 | .000 | .008 | .000 |
| Central | 40 | .601 | .008 | .003 | .024 | .000 | .639 | .010 | .000 | .021 | .000 |
|  | 60 | .689 | .010 | .000 | .019 | .000 | .698 | .017 | .000 | .013 | .000 |
|  | 40+60 | .647 | .010 | .000 | .021 | .000 | .669 | .014 | .000 | .017 | .000 |
| Lateral | 40 | .528 | .022 | .000 | .007 | .014 | .503 | .015 | .000 | .008 | .010 |
|  | 60 | .607 | .016 | .000 | .014 | .000 | .652 | .016 | .000 | .003 | .055 |
|  | 40+60 | .575 | .010 | .000 | .010 | .000 | .574 | .016 | .000 | .005 | .002 |
| Temporal | 40 | .608 | .029 | .000 | .006 | .008 | .601 | .029 | .000 | .013 | .000 |
|  | 60 | .723 | .015 | .000 | .002 | .098 | .746 | .018 | .000 | .004 | .009 |
|  | 40+60 | .673 | .010 | .000 | .004 | .002 | 671 | .023 | .000 | .008 | .000 |
| Parietal | 40 | .579 | .001 | .253 | .015 | .000 | .622 | .004 | .057 | .008 | .003 |
|  | 60 | .633 | .007 | .007 | .010 | .001 | .633 | .017 | .000 | .004 | .036 |
|  | 40+60 | .623 | .010 | .009 | .012 | .000 | .641 | .008 | .000 | .006 | .001 |
